# Activated biochar derived from agricultural and aquatic waste biomass supported on stainless steel mesh for sustainable hydrogen evolution reaction in neutral media

**DOI:** 10.1038/s41598-026-65669-0

**Published:** 2026-08-13

**Authors:** Mervat G. Hassan, Fatma S. Behery, Mahmoud S. A. Shahin, Mohamed O. Abdel-Monem, K. M. El-Khatib, Dena Z. Khater

**Affiliations:** 1 Botany and Microbiology Department, Faculty of Science, Benha University, Banha, Egypt; 2 Main Laboratories for the Egyptian Army, Cairo, Egypt; 3 Chemical Engineering Department, Engineering Research and Renewable Energy Institute, National Research Centre, 33 El-Buhouth St., Dokki, Cairo, 12622 Egypt

**Keywords:** Agricultural waste, Hydrogen evolution reaction, Activated biochar, Neutral pH, Stainless steel mesh, Electrocatalysis, Chemistry, Energy science and technology, Environmental sciences, Materials science, Nanoscience and technology

## Abstract

This work demonstrates a sustainable, scalable, and metal-free approach for converting agricultural waste and aquatic waste biomass (e.g., orange peel (OP), banana peel (BP), rice straw (RS), corn cob (CC), and water hyacinth (WH) into activated biochar while emphasizing the inherent kinetic limitations of hydrogen evolution (HER) in neutral electrolytes. A key challenge in HER under neutral conditions is the sluggish reaction kinetics caused by limited proton availability and slow water dissociation. Comprehensive characterization using X-ray diffraction (XRD), scanning electron microscopy with energy-dispersive X-ray spectroscopy (SEM-EDX), and transmission electron microscopy (TEM) confirmed the formation of activated biochar with a layered carbon structure. Among the investigated materials, RS-, WH-, and CC-derived activated biochar exhibited improved HER performance, requiring overpotentials of 432–436 mV to achieve a current density of 10 mA cm⁻². Electrochemical analysis revealed reduced charge-transfer resistance and increased electrochemically active surface area, contributing to enhanced HER performance. Their corresponding Tafel slopes (323.2–352.4 mV dec⁻¹) are significantly lower than those of OP/SSM (533.5 mV dec⁻¹), BP/SSM (398.2 mV dec⁻¹), and commercial activated carbon as a control (651.67mV dec⁻¹). The enhanced performance is attributed to increased porosity, layered carbon morphology, the presence of intrinsic heteroatoms and mineral phases, improved electrochemically active surface area, and reduced charge-transfer resistance. This work demonstrates a sustainable and metal-free strategy for producing carbon-based electrocatalysts, while highlighting the limitations and challenges associated with HER in neutral electrolytes.

**Supplementary Information:**

The online version contains supplementary material available at 10.1038/s41598-026-65669-0.

## Introduction

The growing global energy demand is strongly linked to continued dependence on fossil fuels, necessitating the development of sustainable energy alternatives^1^. Electrochemical water splitting is widely recognized as a promising route for generating high-purity hydrogen; however, the hydrogen evolution reaction (HER) remains kinetically challenging, particularly under neutral conditions. In neutral electrolytes, HER performance is significantly limited by low proton concentration, slow water dissociation, and unfavorable interfacial kinetics, resulting in higher overpotentials than in acidic or alkaline media^2^. Electrochemical energy technologies, including fuel cells, batteries, and supercapacitors, play a central role in modern renewable energy systems. These technologies enable efficient energy conversion, storage, and distribution, which are essential for integrating intermittent renewable energy sources. In particular, hydrogen serves as both an energy carrier and a storage medium, complementing battery and supercapacitor systems in hybrid energy devices and microgrids^3,4^. Despite these advantages, the development of efficient and cost-effective HER electrocatalysts under neutral conditions remains a major challenge^5^. However, electrochemical water splitting remains the most promising approach for producing high-purity renewable hydrogen. Noble metals, such as platinum, exhibit excellent HER activity but are restricted by their scarcity and high economic cost, which limit large-scale applications^6^. Consequently, considerable research efforts have been directed toward developing low-cost, efficient, and durable alternatives to noble-metal HER electrocatalysts. Among these, carbon-based materials, including graphene^7^, activated carbon^8^, graphene oxide^9^, carbon nanotubes^10^, graphitic carbon^11^ carbon fiber has gained significant attention due to its electrical conductivity, chemical stability, and tunable porosity. Additionally, biomass-derived activated carbon often contains intrinsic heteroatoms (e.g., N, S, P) and mineral components, which can introduce defect sites and modify electronic properties, thereby influencing catalytic performance^12–14^.

Beyond their catalytic properties, biomass-derived materials offer significant environmental advantages. Agricultural and aquatic wastes, such as rice straw, corn cob, fruit peels, and water hyacinth, are produced in large quantities worldwide but are often underutilized or disposed of through environmentally harmful practices, including open burning and landfilling^15^. Converting these abundant wastes into functional carbon materials not only mitigates environmental pollution but also contributes to the development of sustainable energy technologies. This approach aligns with circular economy principles, enabling the transformation of waste into high-value electrocatalysts.

Despite significant advances in biomass-derived carbon electrocatalysts, several challenges remain. First, many studies focus on a single biomass precursor, making it difficult to establish clear relationships between precursor composition, structural properties, and catalytic performance. The variability in biomass composition has been identified as a key limitation affecting reproducibility and performance optimization. Second, most investigations are conducted under strongly acidic or alkaline conditions. In contrast, HER under neutral electrolytes is more relevant for biological systems, wastewater treatment, and microbial electrolysis cells and remains relatively unexplored. Third, high-performing systems often rely on complex synthesis strategies, including heteroatom doping or incorporation of metallic species, which can increase cost and reduce scalability^16^.

While extensive research has focused on acidic and alkaline systems, investigations under neutral conditions remain comparatively limited despite their relevance to practical applications. Therefore, a systematic and comparative investigation of biomass-derived carbon materials under unified synthesis and testing conditions is needed to establish clear structure–property–performance relationships. In particular, understanding how the intrinsic composition and structure of different biomass precursors affect HER performance under neutral conditions is essential for the rational design of sustainable electrocatalysts.

This work provides the first comparative study of activated biochar derived from five abundant biomass feedstocks: orange peel (OP), banana peel (BP), rice straw (RS), corn cob (CC), and water hyacinth (WH). These precursors were selected based on their availability, environmental significance, and compositional diversity, enabling a systematic evaluation of their influence on electrocatalytic behavior. The synthesized activated biochar materials were deposited onto stainless steel mesh and evaluated for HER performance in neutral phosphate-buffered solution and compared with commercial activated carbon (AC) that was employed as a reference. This study aims to (i) establish clear correlations between biomass precursor, structural features, and electrochemical performance; (ii) evaluate HER activity under neutral conditions, where kinetic limitations are more pronounced; and (iii) develop a simple, scalable, and metal-free approach for converting waste biomass into functional electrocatalysts. The findings provide insights into both the potential and limitations of biomass-derived carbon materials for sustainable hydrogen production. This study introduces a scalable approach that leverages waste-derived precursors to produce functional electrocatalysts with reduced environmental and economic cost.

## Materials and methods

Biomass feedstocks (orange peel (OP), banana peel (BP), rice straw (RS), corn cob (CC), and water hyacinth (WH) were collected from local sources in Cairo, Egypt. These materials were selected due to their high availability, environmental relevance, and compositional diversity, enabling systematic comparison of biomass-derived carbon properties. Potassium hydroxide (KOH), ethanol (C_2_H_5_OH, ≥ 99.5%), and polytetrafluoroethylene (PTFE, 60 wt% dispersion) were obtained from Sigma-Aldrich. Nafion (5 wt%) was supplied by the fuel cell store. Stainless steel mesh (SSM) was provided by a company specializing in electronics, science, and technology. All chemicals used in this study were of analytical grade and used without further purification. Ultrapure deionized water (Milli-Q) was used throughout all experiments.

### Fabrication of activated biochar from biowaste materials

The freshly collected OP, BP, RS, CC, and WH were used as biomass precursors for the preparation of activated biochar. The raw materials were separated and washed thoroughly with deionized water to remove soil residues and other unwanted impurities. Then, they were dried at 80 °C for 4 h to remove moisture and fully convert the carbonaceous precursors. The dried samples were ground into a fine powder using a mechanical grinder. This pre-treatment step ensures the removal of contaminants and improves uniformity before thermal processing, as commonly recommended for biomass carbonization^13^. Carbonization was carried out in ceramic crucibles under restricted oxygen conditions by covering the crucibles with lids to minimize air exposure^17^. Each biomass sample was heated in a muffle furnace at 500 °C for 3 h with a heating rate of 15 °C·min⁻¹. The selection of 500 °C was based on achieving a balance between carbon formation and preservation of intrinsic heteroatoms, which were known to contribute to catalytic activity. Higher temperatures typically enhance graphitization but may reduce surface defects and functional groups^18^.

The obtained biochar was chemically activated using KOH at a weight ratio of 1:4 (biochar: KOH). The mixture was dispersed in ~ 50 mL of deionized water and stirred for 2 h to ensure homogeneous impregnation. The slurry was then dried at 105 °C for 12 h before being stored in a desiccator. KOH activation was widely used to enhance the textural porosity, electrical conductivity, and specific surface area in carbon materials through chemical etching and pore formation mechanisms^19,20^. The materials were then washed with 3 M HCl and deionized water to remove residual salts and impurities. Finally, the activated biochar was dried at 80 °C for 12 h and stored for further use.

### Material characterizations

#### Physicochemical analysis

The morphology, elemental composition, and spatial distribution of the prepared activated biochar were analyzed using field-emission scanning electron microscopy (FESEM, JEOL 7000) coupled with energy-dispersive X-ray spectroscopy (EDX). Additionally, the microstructural characteristics were examined using high-resolution transmission electron microscopy (HRTEM, a JEOL GEM-1010). Furthermore, X-ray powder diffraction (XRD) patterns were obtained using the Rigaku Mini Flex 600 to explore the crystallographic phases and chemical bonding characteristics of the sample materials.

### Electrochemical evaluation

#### Working electrodes preparation

The working electrodes for the hydrogen evolution reaction (HER) consisted of a stainless-steel mesh (SSM, 3 × 3 cm²), serving both as a current collector and a supporting structure. The gas diffusion layers of OP, BP, RS, CC, or WH were incorporated on the SSM surface, as previously described^21^. Briefly, activated biochar (25 mg cm⁻²) was mixed with PTFE binder (37.3 mg cm⁻²) and ethanol (approximately 5 mL) to form a uniform paste. The mixture was sonicated in an ultrasonic water bath at 80 °C and then applied onto SSM (3 × 3). The coated electrodes were hot-pressed at 40 MPa and 80 °C for 1 min. The prepared electrodes were labeled as OP/SSM, BP/SSM, RS/SSM, CC/SSM, and WH/SSM. Activated carbon on the SSM (AC/SSM) electrode was also tested under identical conditions to the control sample.

#### Electrochemical characterization

Electrochemical tests were conducted using a three-electrode setup with a Gamry 1010E electrochemical workstation. The produced activated biochar (AB) from the selected biomass loaded on SSM (AB/SSM) was used as the working electrode, Ag/AgCl (3 M KCl) as the reference, and platinum wire as the counter electrode in a 0.1 M phosphate-buffered solution (PBS, pH 7.2). Before measurements, the electrolyte was purged with high-purity nitrogen (N₂) for 20 min to remove dissolved oxygen. Linear sweep voltammetry (LSV) was performed at 5 mV s⁻¹, over a potential range from − 0.5 to -1.3 V (relative to Ag/AgCl), following electrochemical potential activation involving ten CV scans between − 0.46 and − 1.06 V (versus Ag/AgCl) at a scan rate of 100 mV s⁻¹. All potentials were converted to the reversible hydrogen electrode (RHE) scale and corrected for iR drop (measured solution resistance, 23.76, 14.94,13.66,10.14,12.97 and 6.60 Ω for OP, BP, RS, WH, CC, and AC/SSM) using Eq. (1).

1 $$\:{E}_{RHE}={E}_{\left(Ag/AgCl\right)}+0.059\ pH+\:{E}_{\left(Ag/AgCl\right)}^{0}$$

Here, E _Ag/AgCl_ is the measured potential value, E^0^_Ag/AgCl_ = 0.197 V vs. RHE.

The cathodic polarization curves were plotted as overpotential (η) versus log (current density, j) to construct Tafel plots, and the Tafel slope (b) was calculated from the linear region using the equation below.

2 $$\:\eta \: = b\ log{\mathrm{j}}_{{\mathrm{o}}} + a$$

The exchange current density (j₀, mA cm⁻²), which represents the intrinsic HER activity at an overpotential (η) of 0 V, was determined by extrapolating the linear Tafel region to zero overpotential, using the equation j₀ = 10^(a/b). These measurements enable evaluation of the catalyst’s kinetic behavior and facilitate comparisons of HER activity across different activated biochar samples. The electrochemical surface areas (ECSAs) of the produced materials were determined by CV measurements over the potential range of -0.31 and − 0.51 V vs. Ag/AgCl, at scan rates of 20–100 mV s⁻¹, during which only non-Faradaic currents were observed. The capacitance charging current densities were measured at the midpoint of the scan range, and the double-layer capacitance (Cdl, mF cm⁻²) was calculated from the slope of the linear relationship between the charging current density$\:\:\frac{1}{2}$ (ΔJ = Janodic - Jcathodic) and the applied scan rate^22^. The accessible ECSAs for each activated biochar sample were estimated using the following equation:

3 $$\:ECSA= {\mathrm{C/C}}_{{{\mathrm{sp}}}}$$

Where C represents the determined double-layer capacitance, and Csp refers to the specific capacitance, which for the carbonaceous material is considered to be 25 μF cm⁻²^23^. The EIS analysis was performed over a frequency range of 0.1 Hz to 100 kHz, with a polarizing potential of 10 mV and a DC potential of -0.4 V vs. Ag/AgCl. To evaluate the experimental data, the Nyquist and Bode plots were fitted to an equivalent circuit model. The fitting process utilized the simplex method to ensure precise parameter extraction. This methodology offers a quantitative perspective on the interfacial kinetics and emphasizes the impact of catalyst composition and loading density on HER performance. Lastly, Chronopotentiometric measurements were performed at 10 mA cm⁻² for ~ 8000 s (~ 2 h). While this provided an initial stability assessment, longer-term testing was required for practical validation. Therefore, the present results provided preliminary insight into stability.

## Results and discussion

### Fabrication process and characterization

**Fig. 1:**
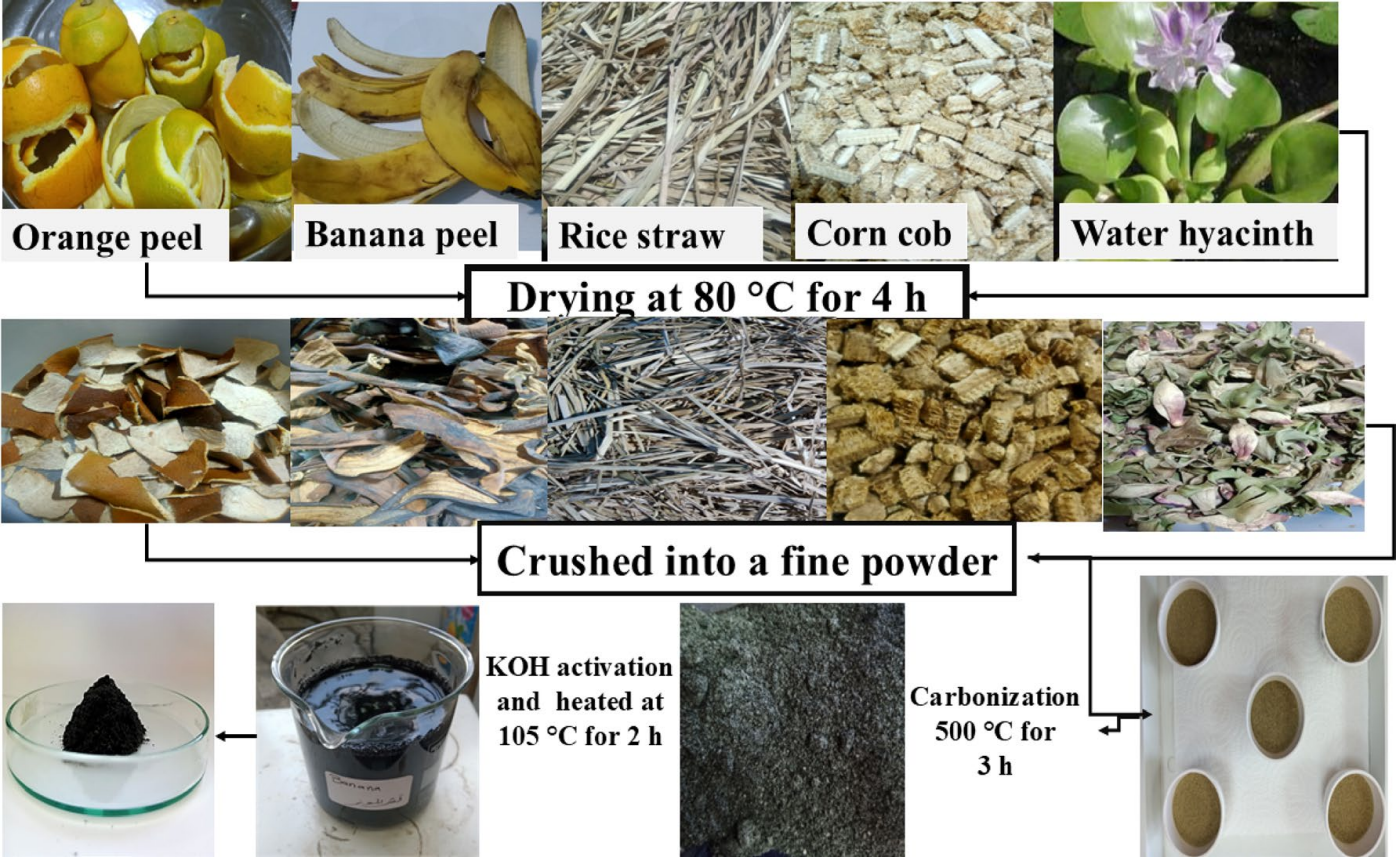
The detailed schematic illustration of the synthesis process for the formation of OP, BP, RS, CC, and WH-activated biomass-derived biochar.

This work presented an effective and novel approach for carbonizing and potassium hydroxide (KOH)–activating a variety of biomass sources to produce high-quality activated biochar. The nominated biomass precursors, orange peel (OP), banana peel (BP), rice straw (RS), corn cob (CC), and water hyacinth (WH), represented plentiful, inexpensive agricultural and aquatic wastes with strong potential for valorization. The experimental portion described the entire process of synthesizing activated biochar (AB) from these resources, which was schematically shown in Fig. 1. It consisted of a series of meticulously regulated procedures intended to optimize the final AB’s performance and structural quality. Each type of biomass was carefully cleaned before processing to remove potential contaminants that could compromise the purity of the final material or hinder effective carbonization. The cleansed biomass was finely powdered to improve homogeneity and surface area. To achieve consistent thermal decomposition and avoid structural flaws during carbonization, the powdered biomass was subsequently dried to remove moisture. The preparation findings demonstrated the great potential for producing valuable carbon materials derived from agricultural and aquatic wastes in a way that is both economically feasible and environmentally sustainable^24^.

During carbonization at 500 °C, the organic components were decomposed and reorganized into a carbon-rich biochar. Subsequently, the chemical activation process using KOH was performed, which was a critical step for generating the high porosity and large specific surface area characteristic of high-performance activated carbon materials. Thus, the adsorptive capability and the accessibility of surface-active sites were significantly enhanced. The obtained activated biochar yield (Y%) from each biomass precursor was calculated to evaluate the material efficiency and assess scalability. The carbonization yields for OP, BP, RS, CC, and WH were 5.35, 20.7, 13.21, 3, and 14.8%, respectively, as shown in Table 1. The structural, morphological, and crystallographic characteristics of the activated biochar samples were thoroughly characterized after carbonization and activation. Porosity, structural integrity, elemental distribution, and crystallinity were evaluated using sophisticated analytical techniques. The microstructural characteristics of each biomass-derived activated biochar and its applicability for electrochemical and catalytic applications were crucially revealed by these analyses.

**Table 1:** Yield percentage of extracted biochar from Agricultural and Aquatic biowastes after carbonization.

Wastes	Biowaste weight before carbonization (g)	Biochar net weight (g)	Yield (%)
Orange peel (OP)	328.5	17.6	5.35
Banana peel (BP)	172.5	35.7	20.7
Rice straw (RS)	51.4	13.21	26
Corn Cob (CC)	96.5	3	3
Water hyacinth (WH)	56	8	14.8

### Physical characterization

#### FE-SEM coupled with EDX

**Fig. 2:**
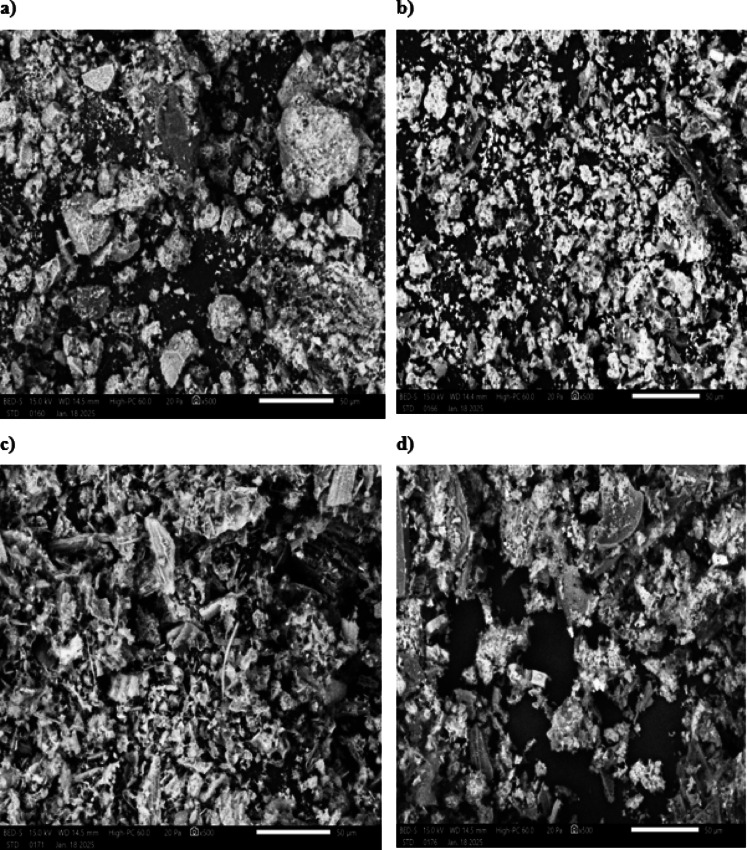

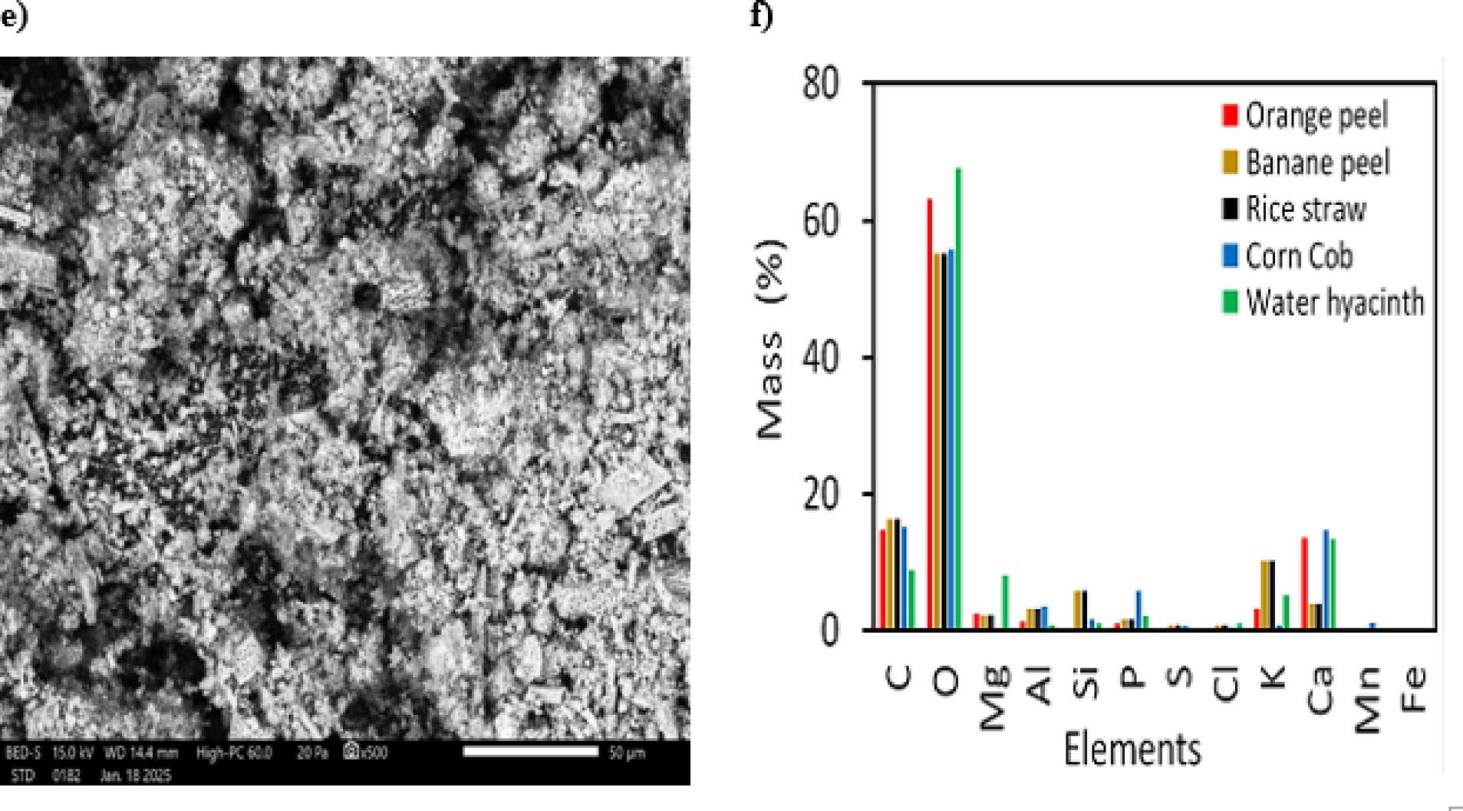
Representative FESEM images of the (**a**) OP, (**b**) BP, (**c**) RS, (**d**) CC, and (**e**) WH, respectively, and (**f**) Essential micronutrients present in activated biomass-derived carbonaceous.

The microstructural features of the OP, BP, RS, CC, and WH activated biochar samples obtained from FE-SEM imaging at different magnifications are shown in Fig. 2(a-f) and Figure S1, and the corresponding EDX spectra and atomic weight percentages are given in Figure S2 and Table S1, respectively. The morphology of all five samples was generally similar, with fractures scattered at random and rough surface textures that suggested structural variability. The samples exhibited uneven, sheet-like domains and tiny aggregated lumps despite their solid bulk. According to EDX analysis, oxygen predominated across all synthesized activated biochars, consistent with the presence of silicates, metal oxides, or surface functional groups. Further, nitrogen, phosphorus, potassium, chlorine, calcium, aluminum, silicon, sulfur, magnesium, and sodium micronutrients were found in trace amounts. Figure 2f summarizes the corresponding mass percentage distribution of these elements, demonstrating the compositional diversity of the activated biochar generated from biomass. FESEM analysis confirmed that the morphological abnormalities were known to improve surface area, which might improve the interaction with electrolytes and boost electrochemical performance^25,26^. Additionally, heteroatoms were known to influence the physicochemical and electrochemical behavior by altering the electronic structure of activated biochar, increasing defect density, and increasing the availability of catalytic sites.

#### HRTEM

**Fig. 3:**
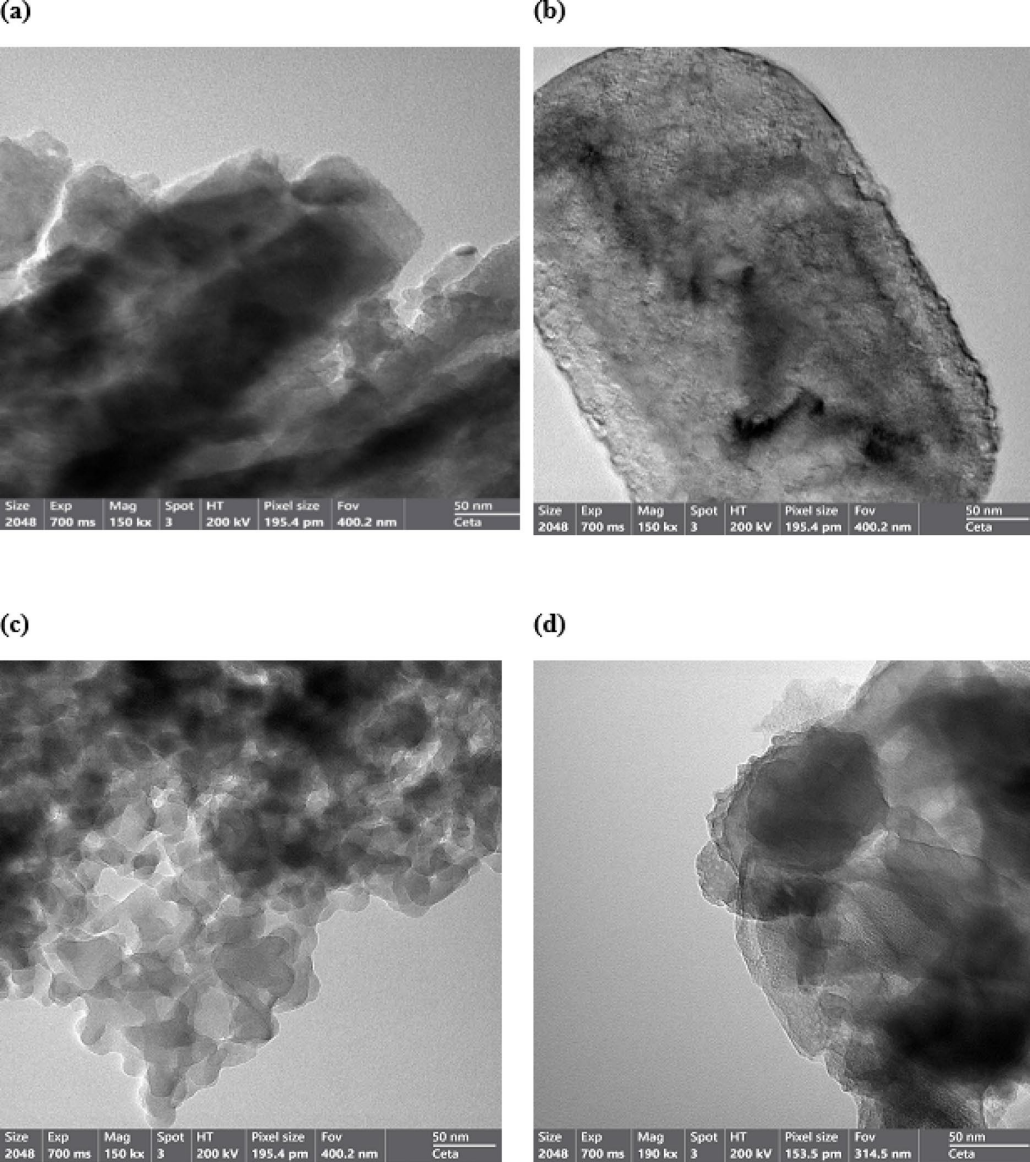

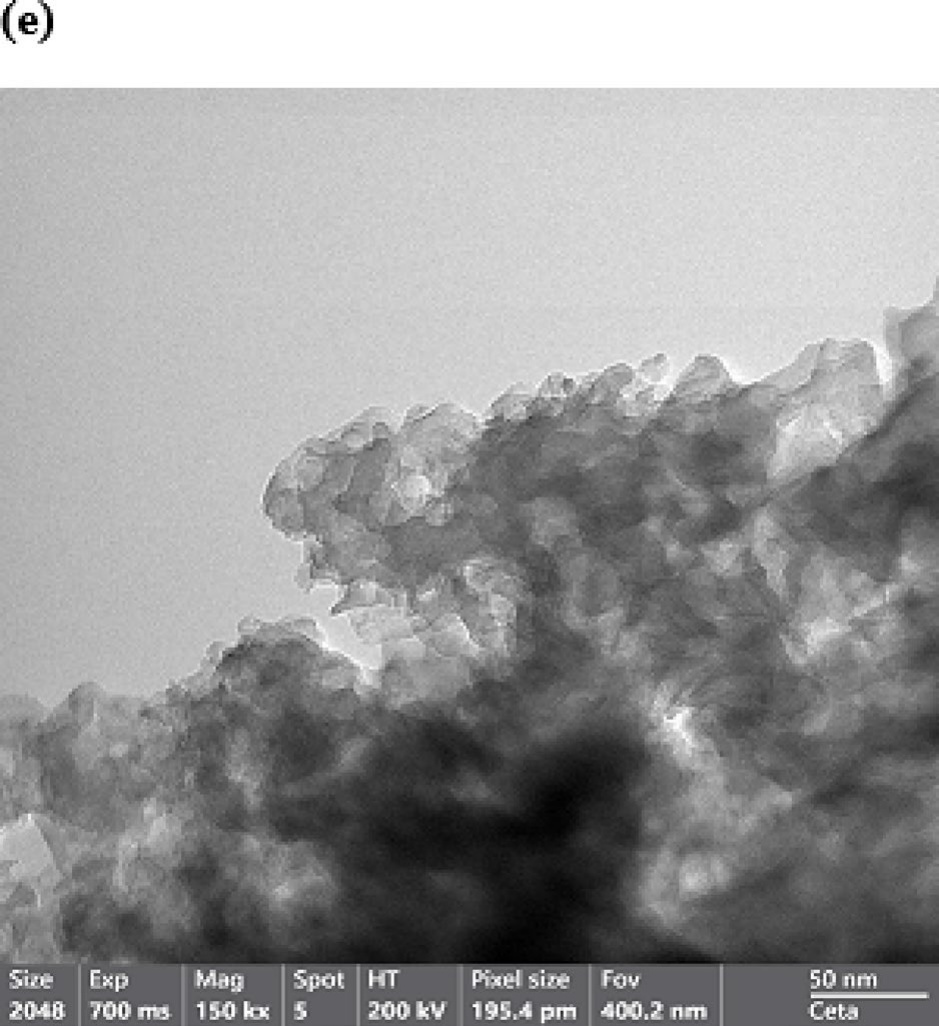
HRTEM images of (**a**) OP, (**b**) BP, (**c**) RS, (**d**) CC, and (**e**) WH activated biochar, respectively.

HRTEM examination further confirmed the presence of layered carbon structures with a graphitic-like structure, as illustrated at different magnifications in Fig. 3(a-e) and Figure S3. Images were captured at magnifications ranging from 150,000 to 245,000X with a scale bar of 50 nm and an operating voltage of 200 kV. The representative HRTEM images revealed striking layered, sheet-like structures formed by irregular carbonaceous agglomerates. The samples as a whole showed discernible differences in density, thickness, and texture due to their different biomass origins. The OP-derived activated biochar was partially transparent with crumpled plate-like layers, as shown in Fig. 3a. The inclusion of thin nanosheets, such as layered carbon structures or graphitic-like areas, was suggested by this translucent behavior^22^. The BP-derived activated biochar, on the other hand, forms capsule-like aggregated or clustered carbon domains with noticeable internal heterogeneity^27^, as seen in Fig. 3b. Internal cavities, variations in structural density, or trapped organic residues could all contribute to this complexity^27^. Densely packed particles with relatively consistent diameters and polygonal to spherical forms were seen in the RS-derived activated biochar (Fig. 3c). The CC-derived activated biochar was shown in Fig. 3d as a compact multilayered flake made of a hierarchical network of thin, stacked sheets. This morphology highlights the structural organization with the aggregation of multilayered carbon structures or graphitic-like areas, underscoring the structural organization within this sample^1^. Finally, the WH-derived activated biochar, which has sheet-like areas scattered with granular, rough, or fragmented carbonaceous clusters, was shown in Fig. 3e. These results provided important insights into the possible electrochemical and catalytic behavior of the activated biochar materials derived from the five biomass sources, highlighting their diversity and complexity.

#### XRD

**Fig. 4:**
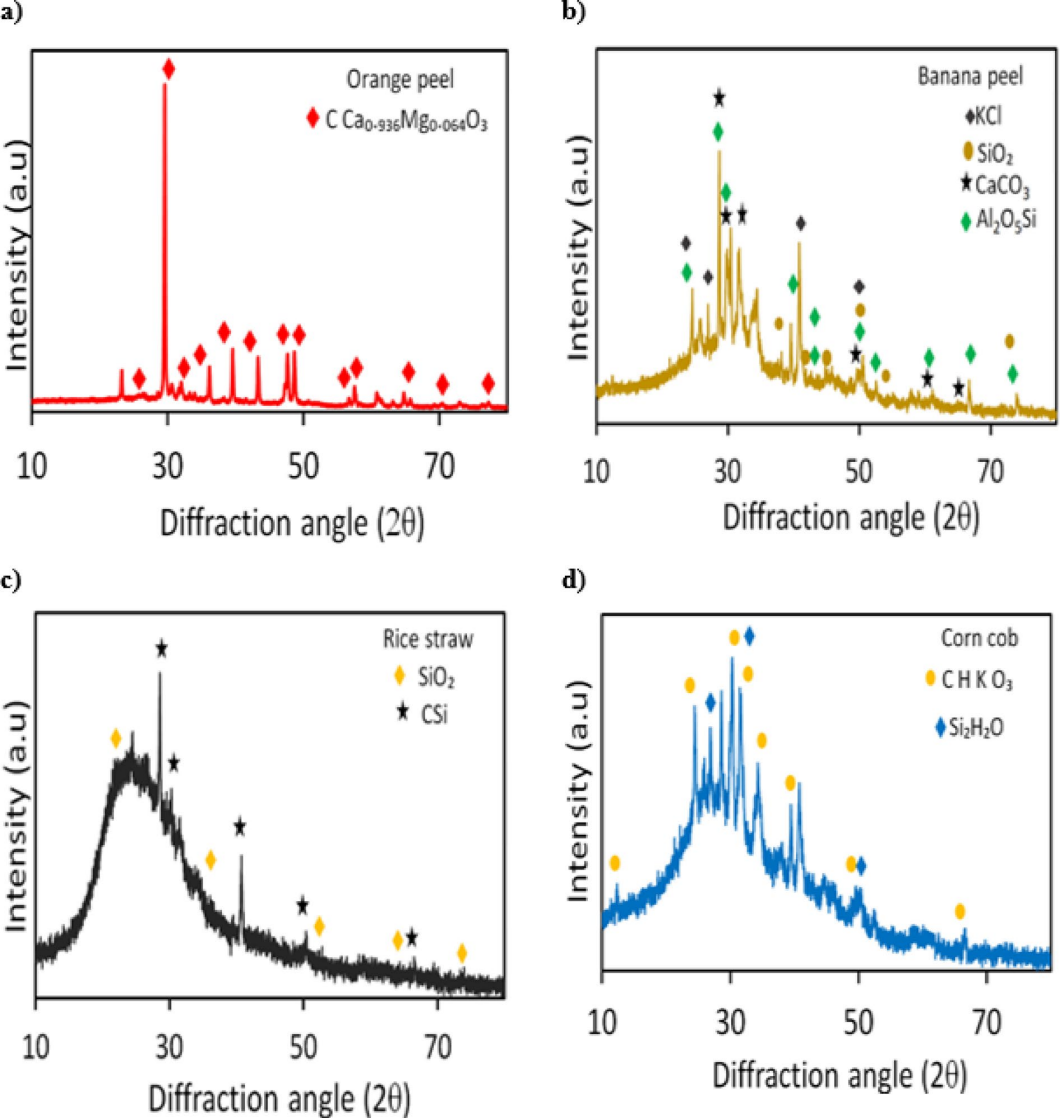

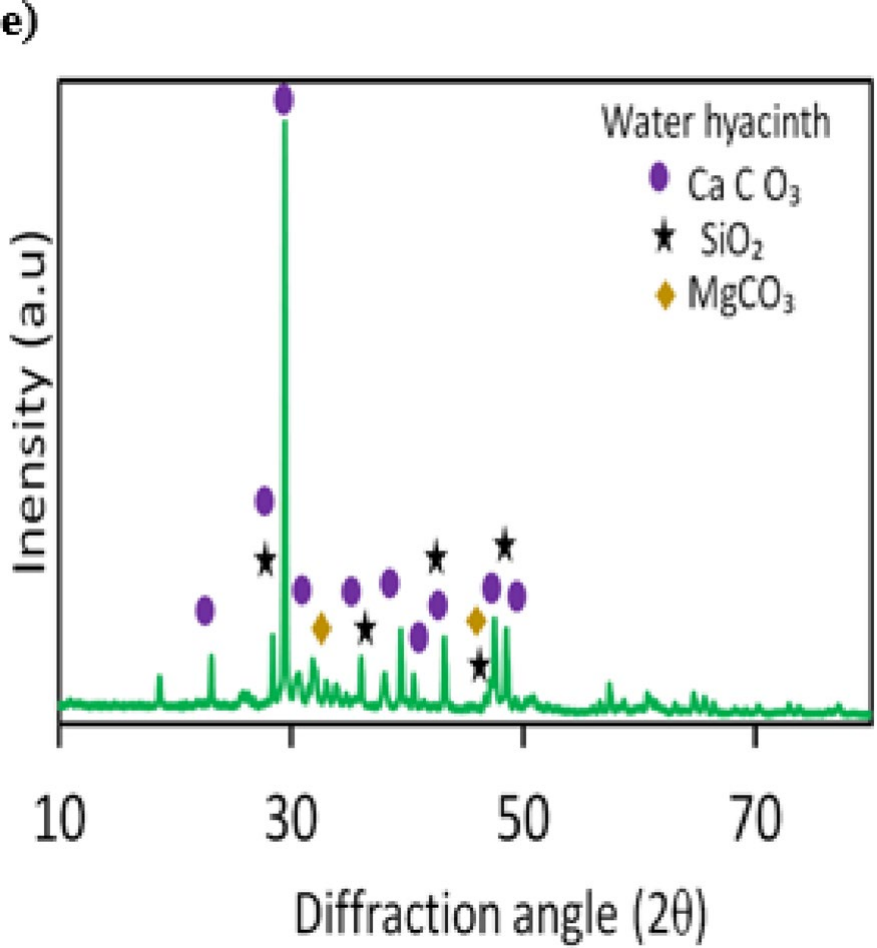
(**a**–**e**) XRD patterns of the as-prepared activated biochar in the carbon framework for OP-, BP-, RS-, CC-, and WH- activated biochar, respectively.

The XRD patterns presented in Fig. 4 provided insights into the crystallographic structure and composition of various activated biochar samples derived from different agricultural wastes. Each subfigure 4(a-e) displays a specific type of biochar, with distinct peaks indicating the presence of specific mineral phases or crystalline structures. It could be noticed that all biochar samples exhibited distinct crystalline mineral phases, which could be attributed to the homogeneous distribution of metals within the biochar matrix, leading to a lack of separate carbon peaks that would be completely masked by inorganic metals. The presence of these phases further confirms the mineral-rich nature of the produced biochar, which aligns with findings that reported similar mineral phases in biochar derived from agricultural residues^28^.

The XRD pattern of OP-derived activated biochar (Fig. 4a) showed, well-defined peaks, particularly at 2θ values of 23.21°, 29.54°, 31.53°, 36.10°, 39.54°, 43.30°, 47.23°, 47.62°, 48.62°, 56.69°, 57.51°, 58.20°, 60.78°, 61.08°, 61.46°, 63.15°, 64.75°, 72.94°, 76.41°, and 77.22°, consistent with JCPDS#9,001,297. The dominance of these peaks suggested that the OP biochar contains a significant amount of inorganic minerals, likely due to the high ash content in the original biomass^29^. The presence of Ca₀.₉₃Mg₀.₀₆₄O₃ further confirmed the mineral-rich nature of this biochar.

The XRD pattern for BP-derived activated biochar (Fig. 4b) exhibited a broad and less intense peaks at 2θ around ≈ 24.59°, 26.99°, 29.84°, 31.72°, 39.59°, 40.85°, 40.85°, 45.82°, 50.49°, 52.54°, 55.15°,59.01°, 60.97°,66.68°, and 73.96°, along with sharper peaks at higher angles. The identified phases include SiO₂ (JCPDS#1011176), KCl (JCPDS#9003117), CaCO₃ (JCPDS#1010928) and Al_2_O_5_Si (JCPDS#9000719). The broad peak suggested the presence of amorphous carbon, while the sharper peaks indicated crystalline mineral phases. The combination of amorphous and crystalline structures implies that BP-derived activated biochar may have a heterogeneous composition, with both organic (carbonaceous) and inorganic (mineral) components. The RS-derived activated biochar exhibited a broad amorphous hump between 28.556 and 40.693° (Fig. 4c), indicating partially disordered carbon with low crystallinity^30^. Crystalline peaks corresponding to SiO₂ (JCPDS#1536409) were superimposed. The sharpness and intensity of these peaks suggest a highly crystalline structure, likely due to the silica-rich composition of rice straw^31^. The presence of SiO₂ was particularly notable, as it was a common component in plant-based biochar and can contribute to thermal stability and mechanical strength^30^.

As shown in Fig. 4d, the CC-derived activated biochar exhibited multiple peaks across the 2θ range, with notable crystalline phases associated with CHKO₃ (JCPDS#9016304) and hydrated silica (Si₂H₂O, JCPDS#1536253). The distribution of peaks suggested a mixed crystalline and amorphous structure, with the potassium-based compounds likely contributing to the ionic conductivity of the biochar. The presence of silicon-based phases further indicated the retention of inorganic minerals from the original biomass. Meanwhile, multiple crystalline phases corresponding to CaCO₃ (JCPDS#9016706), SiO₂ (JCPDS#1536409), and MgCO_3_ (JCPDS#9001849) were observed in the WH-derived charcoal (Fig. 4e). The high intensity of the CaCO₃ peak suggests that calcium carbonate is the primary crystalline phase in this biochar. The presence of MgCO₃ and SiO₂ further indicated a mineral-rich composition.

Residual inorganic phases (e.g., silicates or carbonates) were detected in some samples. These phases may arise from the intrinsic mineral content of the biomass precursors. While these species can influence surface properties such as wettability and defect formation, they are not considered primary active sites for HER but may indirectly affect catalytic behavior^18^. According to XRD measurements, SiO₂ was present in all samples except OP, indicating the original biomasses’ mineral composition^32^. The coexistence of diverse mineral phases within the activated biochar might positively influence their electrocatalytic behavior by introducing active sites, modulating electronic properties, and promoting synergistic interactions with the carbon matrix. These structural features may facilitate electron transport and improve accessibility to catalytically active sites, thereby contributing to enhanced HER performance^33^. Therefore, the XRD results confirmed the successful synthesis of activated biochar from agricultural residues and aligned well with the morphological features observed in FESEM and HRTEM analyses.

### Electrochemical characterization

#### HER analyses

**Fig. 5:**
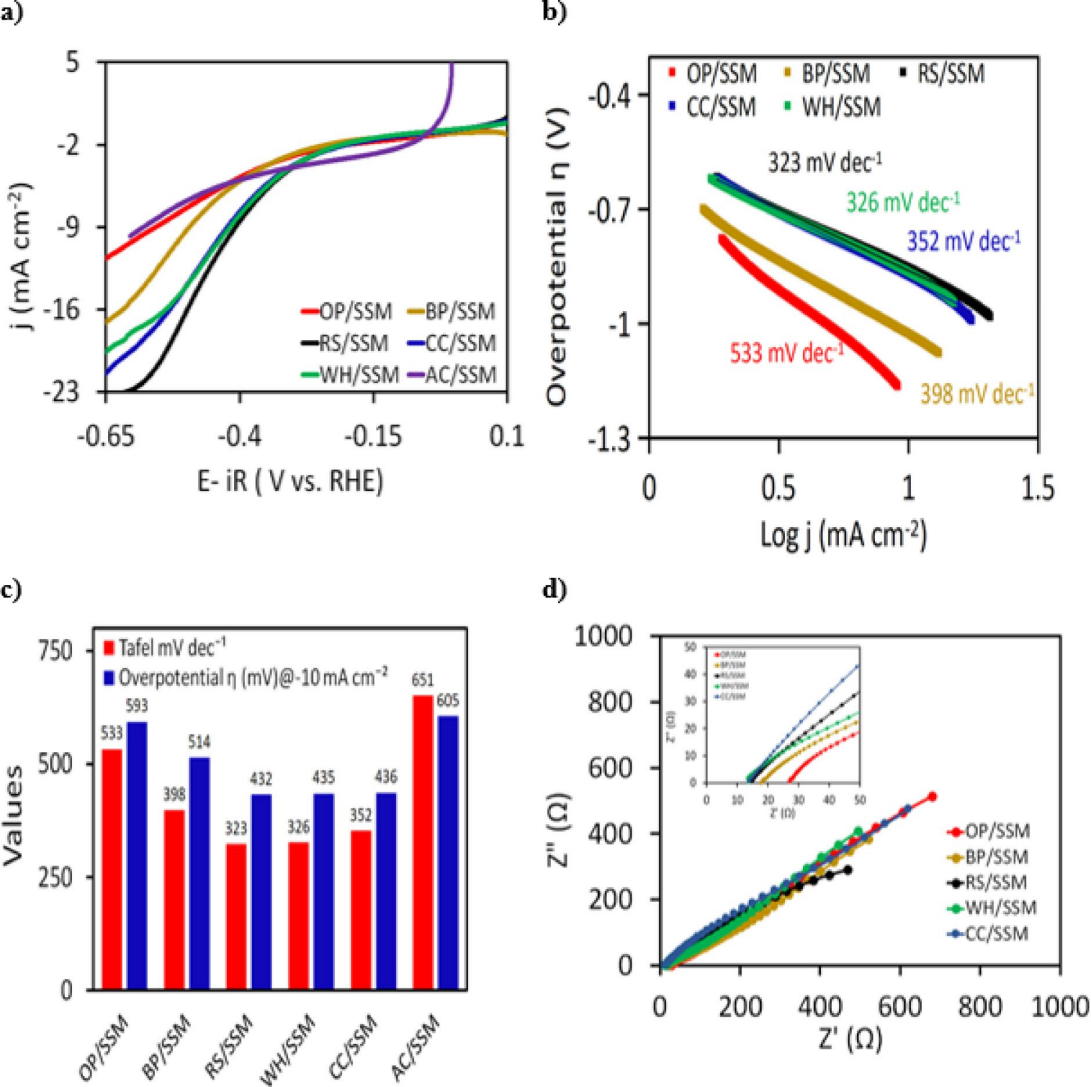

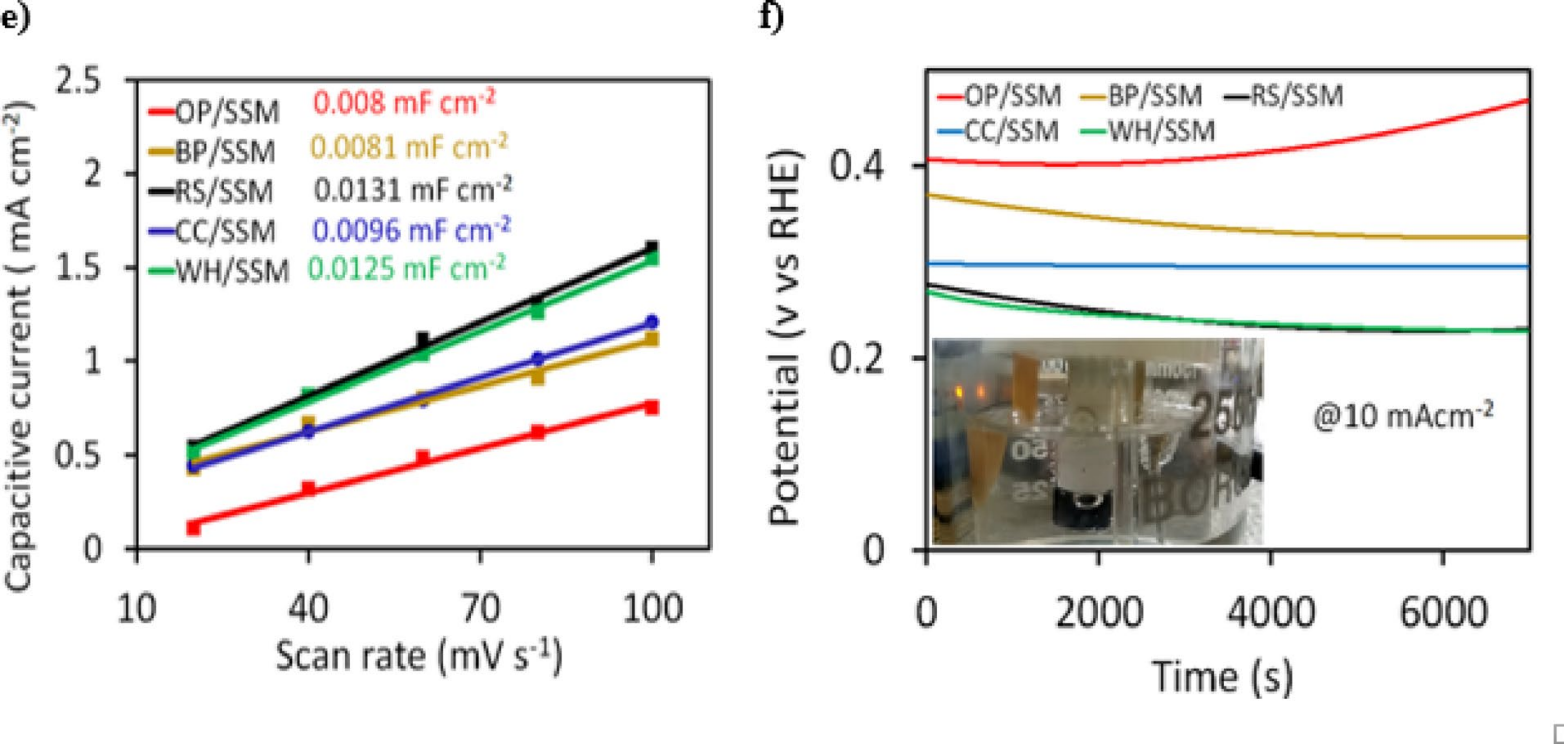
(a) HER LSV polarization curves at a scan rate of 5 mV s^−1^ in 0.1 M PBS (E-iR corrected), (**b**) Corresponding Tafel plots with the corresponding Tafel slopes (mV dec⁻¹) for the HER process, (**c**) The required overpotentials to achieve 10 mA cm^-2^, (**d**) EIS, (**e**) Cdl plots (linear relationship of charging current density against the applied scan rate ranging from 20–100 mV s^-1^, and (**f**) Chronopotentiometric durability test at constant current density of10 mA cm^-2^ for OP/SSM, BP/SSM, RS/SSM, CC/SSM and WH/SSM electrodes in neutral pH, where the inset shows the optical images of H_2_ bubbles generated on the electrode surface on the HER currents.

The HER electrocatalytic activity of the synthesized activated biochar (AB) on the SSM (AB/SSM) series was initially evaluated using a three-electrode configuration in 0.1 M PBS (pH 7.2). The linear sweep voltammetry (LSV) polarization curves were displayed as current density (j, mA cm⁻²) versus iR-corrected potential (E-iR vs. RHE). For comparison, commercial AC/SSM was also measured under the same circumstances. As illustrated in Fig. 5a, the electrocatalytic HER performance of the prepared activated biochar in this study varied substantially with biomass source. Among the investigated samples, RS/SSM exhibited the best overall activity, delivering the highest current density (23.507 mA cm⁻²) and the lowest overpotential (η_10_) of 432 mV at 10 mA cm⁻² among the five prepared catalysts, indicating more favorable charge-transfer kinetics. CC/SSM showed comparable behavior, achieving a current density of 21.676 mA cm⁻² with an overpotential of 436 mV. WH/SSM also demonstrated similar activity, maintaining a current density above 20 mA cm⁻² and a lower η_10_ of 435 mV. In contrast, BP/SSM, OP/SSM and particularly AC/SSM exhibited inferior performance, characterized by markedly lower current densities (17.248, 11.799, and 10.062 mA cm⁻², respectively), higher overpotentials (514–600 mV), reflecting less efficient HER electrocatalysis.

This highlights that the present materials exhibit moderate but competitive performance within the context of neutral HER systems^34^. The results demonstrated that integrating activated biochar with stainless steel mesh electrodes could enhance HER activity under neutral conditions. To further investigate the HER kinetics, the corresponding Tafel analysis (Fig. 5b and Figure S4) was carried out. The Tafel slopes for RS/SSM, WH/SSM, and CC/SSM were found to be in the range of 323.2-352.4 mV dec⁻¹ by fitting the linear region of the η versus log(j) plot. These values were significantly higher than those reported for noble-metal catalysts but were consistent with metal-free carbon systems operating in neutral electrolytes. Compared to OP/SSM (533.5 mV dec⁻¹), BP/SSM (398.2 mV dec⁻¹), and AC/SSM (651.67 mV dec⁻¹), these values were significantly lower. A comparative summary of Tafel slope and overpotentials for the activated biochar-derived electrodes was presented in Fig. 5c.

The relatively high Tafel slopes indicate sluggish reaction kinetics, suggesting that the electrochemical desorption step (Heyrovsky reaction) is rate-limiting. This behavior is expected under neutral conditions, where proton availability is limited and water dissociation becomes the dominant pathway for hydrogen generation. Consequently, the reaction requires additional energy to activate water molecules, leading to higher overpotentials than in acidic systems. The HER proceeds through a sequence of elementary reactions, typically involving electrochemical desorption (Heyrovsky step)^35^.

4 $$\:{H}_{ads}{+\:H}_{2}O+{e}^{-}\leftrightarrow\:{H}_{2}+O{H}^{-}+* \;\;\;\;\;\;{\text{(Heyrovsky step)}}$$

where * denotes an unoccupied active site on the surface of activated biochar.

The HER under neutral electrolyte conditions presents intrinsic kinetic limitations compared to acidic or alkaline media. In neutral phosphate-buffered solutions, the concentration of freely available protons is significantly lower; therefore, hydrogen generation primarily relies on water dissociation rather than direct proton reduction. This process is inherently slower and requires additional activation energy, leading to higher overpotentials and reduced reaction rates. Furthermore, the buffering action of the electrolyte plays a dual role by maintaining stable pH conditions while simultaneously limiting rapid proton availability at the electrode–electrolyte interface. As a result, proton transport toward catalytically active sites becomes restricted, further slowing HER kinetics. These combined factors, including sluggish water dissociation, limited proton transport, and buffer-mediated constraints, contribute to the overall difficulty of achieving efficient HER activity under neutral conditions. Consequently, the relatively high Tafel slopes and moderate catalytic performance observed in this study can be attributed primarily to these inherent limitations rather than solely to the material properties^36–38^.

The exchange current density (j₀, mA cm⁻²), a crucial measure derived by extending the Tafel plots to an overpotential of 0 V vs. RHE, was used to further evaluate the materials’ intrinsic catalytic activity. For RS/SSM, WH/SSM, and CC/SSM, the computed j₀ values were 2.16 × 10^− 2^, 2.18 × 10^− 2^, and 2.33 × 10^− 2^ mA cm⁻², respectively. The improved intrinsic HER activity of the RS/SSM, WH/SSM, and CC/SSM electrodes was confirmed by these values, which were much greater than those found for OP/SSM (6.33 × 10^− 2^ mA cm⁻²), BP/SSM (2.6 × 10^− 2^ mA cm⁻²), and commercial AC/SSM (4.37 × 10^− 2^ mA cm⁻²), confirming the improved intrinsic HER activity of the RS/SSM, WH/SSM, and CC/SSM electrodes. The low j₀ confirmed limited intrinsic catalytic activity, indicating that although the materials provide accessible active sites, the binding energy of hydrogen intermediates and reaction kinetics remain suboptimal. However, the intrinsic catalytic activity remains moderate, as reflected by the low exchange current density, highlighting the need for further material optimization.

#### EIS analysis

EIS measurements were carried out before the durability test in a neutral 0.1 M PBS to obtain a fuller understanding of the kinetics driving the remarkable HER activity and the charge transfer behavior at the AB/SSM-electrolyte interface. The Nyquist plots were displayed in Fig. 5d, and the corresponding circuit utilized for fitting was displayed in Figure S5. OP/SSM had the slowest charge-transfer resistance (Rct) at 2.57 Ω and the largest impedance response among the evaluated AB/SSM electrodes, indicating the worst HER kinetics. RS/SSM, WH/SSM, and CC/SSM, on the other hand, showed the smallest arc, which corresponded to much reduced Rct of 1.09, 1.29, and 1.37 Ω, respectively. Moreover, all electrodes exhibited an inclined semicircle, confirming that Rct controls the HER kinetics, while mass-transport constraints were insignificant under the measurement conditions^27^. As summarized in Table S2, the electrolyte resistance (R_s_) values were nearly identical for all samples, whereas the R_ct_ varied significantly. This observation indicates that the impedance differences primarily originate from interfacial processes at the AB/SSM electrode–electrolyte interface rather than from variations in electrolyte resistance. Reduced Rct indicates enhanced interfacial electron transfer, which contributes to improved HER performance^39^. The correlation between lower Rct and lower overpotential suggested that charge transfer kinetics play a dominant role in determining catalytic activity under neutral conditions^40^.

Compared with OP/SSM, the remaining electrodes showed intermediate semicircle diameters, reflecting progressive enhancements in electrical conductivity, water activation capability, accessibility of active sites, hydrogen intermediate (H*) adsorption/desorption behavior, and interfacial wettability. Moreover, AC/SSM (Figure S6) displayed the lowest Rct value among all evaluated materials. This discrepancy might be attributed to hydrogen bubble accumulation, sluggish surface kinetics, increased energy requirements, and related mass transport boundaries at the AC/SSM electrolyte interface. The overall HER activity was as follows: RS/SSM > WH/SSM > CC/SSM > BP/SSM > OP/SSM.

Furthermore, the electrochemical surface areas (ECSAs) in the non-Faradaic region were evaluated using cyclic voltammetry at scan speeds of 20–100 mV s⁻¹ to further compare the electrocatalytic activity across samples obtained from AB (Figure S7). The ECSAs, calculated from the slope of the capacitive current versus scan rate plot representing the double-layer capacitance (Cdl), were presented in Fig. 5e. Compared with OP (3.20 cm²) and BP (3.24 cm²), the RS-, WH-, and CC-derived activated biochar samples exhibited a greater density of accessible active sites, with values of 5.24, 5.0, and 3.84 cm², respectively. This was consistent with their more developed porous morphology observed in FESEM images. However, it should be noted that ECSA values were based on an assumed specific capacitance value of 25 µF cm⁻², which is commonly reported for carbon-based materials.

The improved HER performance of RS-, WH-, and CC-derived activated biochar can be attributed to a combination of structural and electrochemical factors, such as higher porosity and surface roughness, improved electron transport pathways and intrinsic heteroatoms and mineral phases. This synergistic effect was consistent with previous studies showing that biomass-derived carbon materials exhibit enhanced catalytic behavior when structural defects, porosity, and surface functionalities are optimized^39^. Residual inorganic phases such as CaCO₃ and SiO₂ were detected in the biochar samples. These species may influence the catalytic performance indirectly by modifying surface defects, electronic structure, or wettability. To better position the present results, the HER performance was compared with previously reported biomass-derived catalysts in neutral media (Table 2). It was noteworthy that the prepared activated biochar electrocatalysts were unable to sustain the current densities required at 20, 50, and 100 mA cm⁻², indicating limitations in their intrinsic HER activity under the investigated conditions. Therefore, further optimization through the incorporation of transition-metal oxides or other catalytic species was recommended to enhance electrocatalytic activity, electrical conductivity, and charge-transfer kinetics, as widely reported in previous studies^18,24,27,30,32,5,41–44^. Although the overpotential values obtained in the present work were generally higher than, or close to, those reported for advanced biomass-derived electrocatalysts, they remain comparable to many undoped biomass-based carbon materials evaluated under neutral electrolytes, where overpotentials frequently range from approximately 320 to 600 mV^32,39,43,45,46^.

The relatively low current densities observed in this study may be attributed to the inherent complexity of HER in neutral media, where sluggish water dissociation and multiple reaction pathways can limit reaction kinetics and increase energy barriers. Consequently, despite demonstrating measurable electrocatalytic activity, the prepared biochar exhibited only moderate HER performance compared with several previously reported biochar-based electrocatalysts^39,46^. Nevertheless, the results clearly demonstrate that the choice of biomass precursor plays a critical role in determining electrocatalytic behavior, with agricultural residues as counterparts investigated in this work. These findings emphasize the importance of precursor selection and provide a foundation for future catalyst improvement through compositional and structural engineering.

**Table 2:** Comparison of biomass-derived HER electrocatalysts in neutral, acidic and alkaline media.

Electrocatalyst	Biomass Source	Electrolyte	J, (mA cm⁻²)	η_10_ (mV)	Tafel Slope (mV dec⁻¹)	Ref.
RS/SSM	Rice straw	PBS	23.507	432	323.2	This study
WH/SSM	Water hyacinth	PBS	20.103	435	326.8	This study
CC/SSM	Corn cob	PBS	21.676	436	352.4	This study
BP/SSM	Banana peel	PBS	17.248	514	398.2	This study
OP/SSM	Orange peel	PBS	11.799	593	533.5	This study
Biochar-modified electrode	Cattle manure	KOH	0.2	~ 320	25.7	^45^
Waste-derived carbon catalyst	S. rubra	H_3_PO_4_ and KOH	6–16	~ 550	147	^47^
Mo_2_C nanostructures	Birch forestry residue (biochar)	M H_2_SO_4_	10–100	35	25	^46^
HH-AC-700 layered nanobundles	Human hair	M H_2_SO_4_	120	16	51	^27^
SNC(C)	Sunflower seed shells	PBS	~ 5	450	-	^41^
CFAC/Ni&Co oxides	Chicken Feathers	M KOH	80	87	50	^42^
RN-800	Peanut root nodules	M H_2_SO_4_	100	116	67.8	^43^
CoP@NSPC	Ginkgo leaves	M H_2_SO_4_	60–100	160	87.3	^44^
yolk-shell Co-CoO/BC	Holly leaves	KOH	40	210	93.3	^48^
NiOx-AC-500	Cauliflower leaves	M KOH	40–50	180	121	^32^
CoP/C	Magnolia leaves	M KOH	40–200	140	109.30	^30^
Ni-doped graphitic carbon	White rose petal	M KOH	40–50	497	77	^16^
WO_3_/B-AC	Neem leaves	M KOH	120	360	14	^5^
HPNS	Palm spathe-pollen	M KOH	20	330	63	^49^
Chilli-Ni 800	Chilli Fruits	M KOH	40–50	410	-	^24^
Tea waste	Tea waste	NaOH H_2_SO_4_	3–5	374	44.9	^50^
N-, P- and Ca-codoped	Pig bones	H_2_SO_4_	80	162	80	^51^

#### Stability assessment

Chronopotentiometric tests demonstrated a stable current density of 10 mA cm⁻² versus RHE over ~ 8000 s (~ 2 h) with minimal potential drift for the best-performing electrodes. This test simulated prolonged operational conditions, as evidenced by optical images showing continuous hydrogen evolution on the activated biochar-coated SSM electrode surface under neutral conditions. Throughout the experiment, all AB/SSM electrodes showed continuous hydrogen bubbling. The RS/SSM, WH/SSM, and CC/SSM electrodes revealed moderate but competitive catalytic performance under neutral conditions throughout the test, as shown in Fig. 5f. They maintained nearly constant potentials of about 0.30 V vs. RHE with very little drift.

These observations are consistent with the improved HER activity of RS/SSM, WH/SSM, and CC/SSM and demonstrate their relatively stable operation during the short-term durability test conducted under neutral conditions. Whereas BP/SSM exhibited moderate behavior, characterized by a slight downward trend in potential, reflecting electrochemical activation through improved electrolyte wetting and/or the exposure of additional active sites. In contrast, OP/SSM displayed the largest potential fluctuations, suggesting ineffective charge transfer, unstable interfacial kinetics, and possible surface restructuring. These results highlighted the critical role of efficient charge transfer kinetics in ensuring short-term (~ 2 h) HER operation in neutral electrolytes; however, this duration is insufficient to confirm long-term durability for practical applications. Therefore, the present results provide preliminary insight into stability, and extended testing is required in future work.

## Conclusion and future perspectives

This study demonstrates a simple and scalable approach for converting diverse agricultural and aquatic waste materials into activated biochar for the hydrogen evolution reaction (HER) under neutral conditions. A systematic comparison of five biomass precursors: orange peel, banana peel, rice straw, corn cob, and water hyacinth. Among the investigated samples, RS-, WH-, and CC-derived biochar exhibited relatively improved HER performance, achieving overpotentials of 432–436 mV at 10 mA cm⁻². These materials also showed lower charge-transfer resistance and higher electrochemical surface area, which contributed to enhanced catalytic behavior. The improved HER performance was attributed to the synergistic effects of increased porosity, layered carbon morphology, the presence of intrinsic heteroatoms and mineral phases, improved electrochemically active surface area, and reduced charge-transfer resistance. However, the electrocatalytic activity remains moderate compared with state-of-the-art HER catalysts. The relatively high Tafel slopes (> 200 mV dec⁻¹) and low exchange current densities indicate sluggish reaction kinetics, which are primarily governed by the limitations of HER in neutral electrolytes. Future work should focus on improving intrinsic activity through heteroatom doping or hybrid catalyst design, alongside advanced characterization (e.g., XPS, Raman) to identify active sites. Optimization of pore structure, conductivity, and surface chemistry will be crucial for enhancing performance. In addition, extended stability testing (≥ 24 h), evaluation at higher current densities, and testing in practical device configurations are necessary for real-world validation. Finally, systematic optimization of synthesis conditions and scalability assessment will be essential to realize practical applications of biomass-derived electrocatalysts. Thus, this work highlights that biomass-derived activated biochar can serve as a sustainable and low-cost platform for HER electrocatalysis in neutral media. At the same time, the results emphasize the inherent kinetic challenges of neutral HER and the need for further material optimization to achieve higher catalytic performance.

## Supplementary Information

Below is the link to the electronic supplementary material.

Supplementary Material 1

## Data Availability

The authors declare that the data supporting the findings of this study are available within the paper and its Supplementary Information files.
