# Supplementary material for: Activated biochar derived from agricultural and aquatic waste biomass supported on stainless steel mesh for sustainable hydrogen evolution reaction in neutral media
Source: Sci Rep. 2026 Aug 13;16:25197. doi: 10.1038/s41598-026-65669-0 (PMC13469548; doi:10.1038/s41598-026-65669-0)
Supplement: Supplementary file 1 [file 41598_2026_65669_MOESM1_ESM.pdf]

Supplementary information

Activated Biochar Derived from Agricultural and Aquatic Waste Biomass Supported on Stainless Steel Mesh for Sustainable Hydrogen Evolution Reaction in Neutral Media

Mervat G. Hassan, [1] Fatma S. Behery, [1] Mahmoud S.A. Shahin, [2] Mohamed O. Abdel-Monem, [1] Kamel M. El-Khatib, [3] and Dena Z. Khater*, [3]

[1] Botany and Microbiology Department, Faculty of Science, Benha University, Egypt

[2] Main laboratories for the Egyptian Army, Egypt

[3] Chemical Engineering Department, Engineering Research and Renewable Energy Institute, National Research Centre, 33 El-Buhouth St., Dokki, Cairo, 12622, Egypt

*** Corresponding author**

Dena Z. Khater

Emails: dz.khater@nrc.sci.eg , dena_khater2001@yahoo.com

Figure No. (7)

Table No. (3)

Activated biochar	
**Orange Peel (OP)**	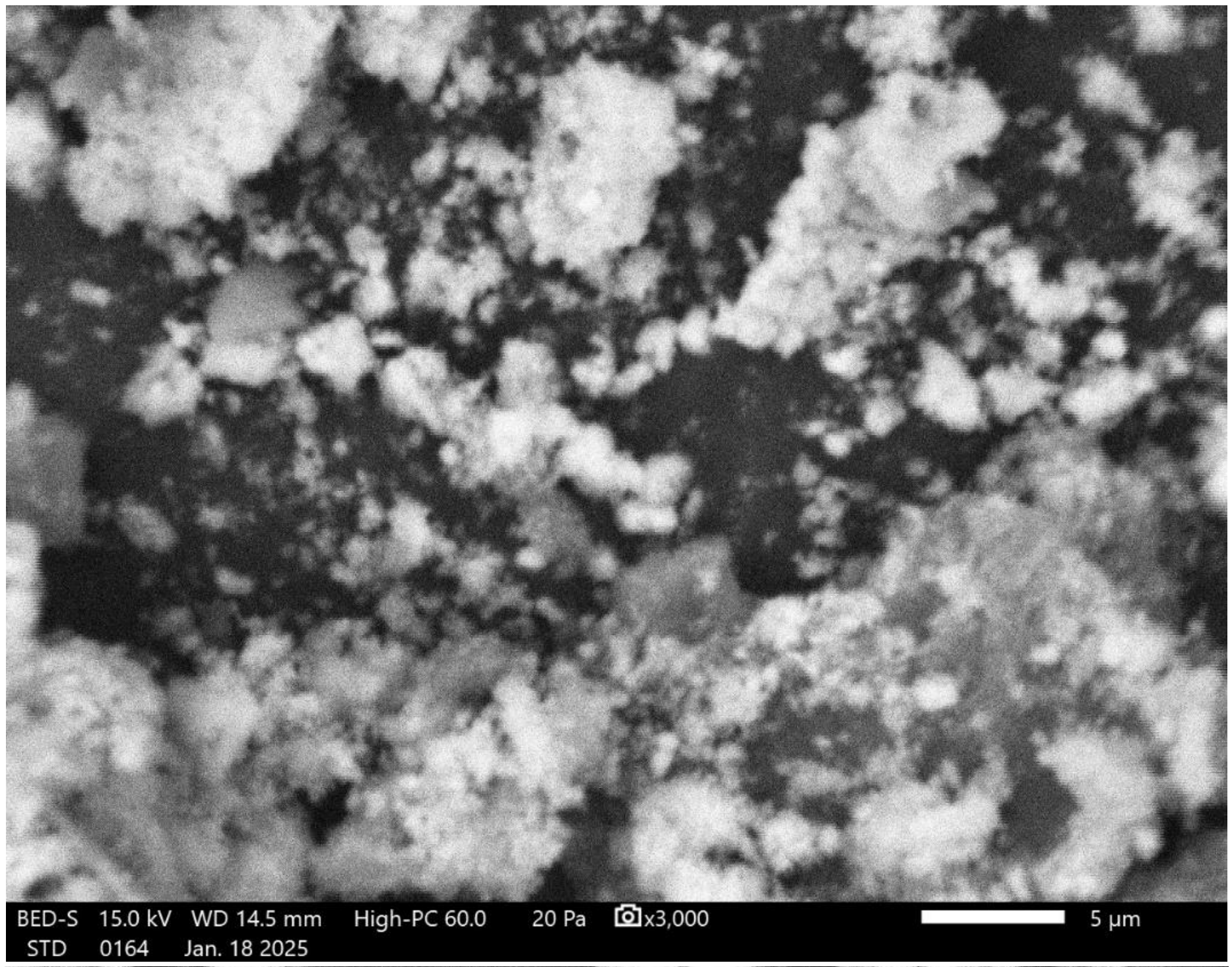
**Banana Peel (BP)**	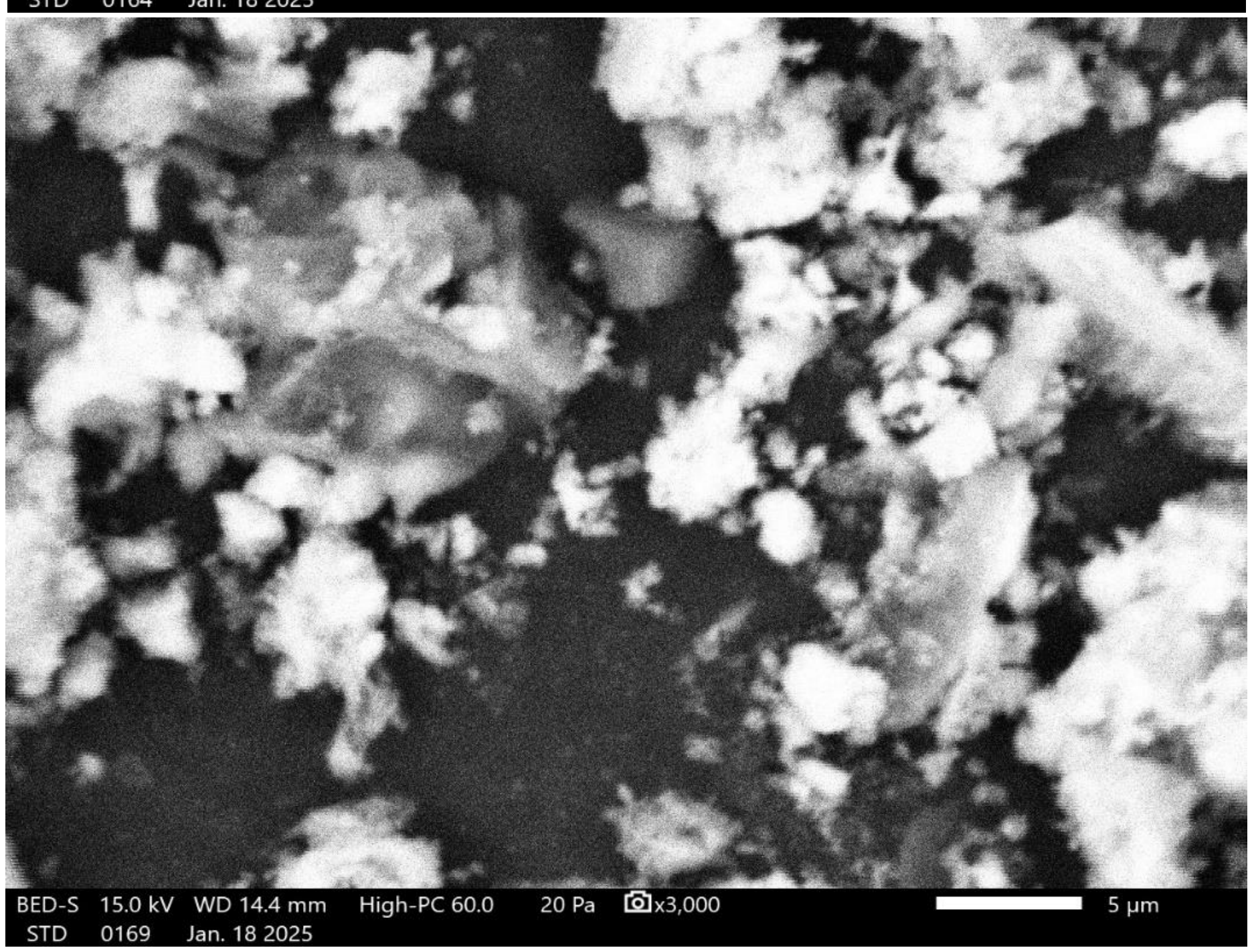

**Rice Straw (RS)**	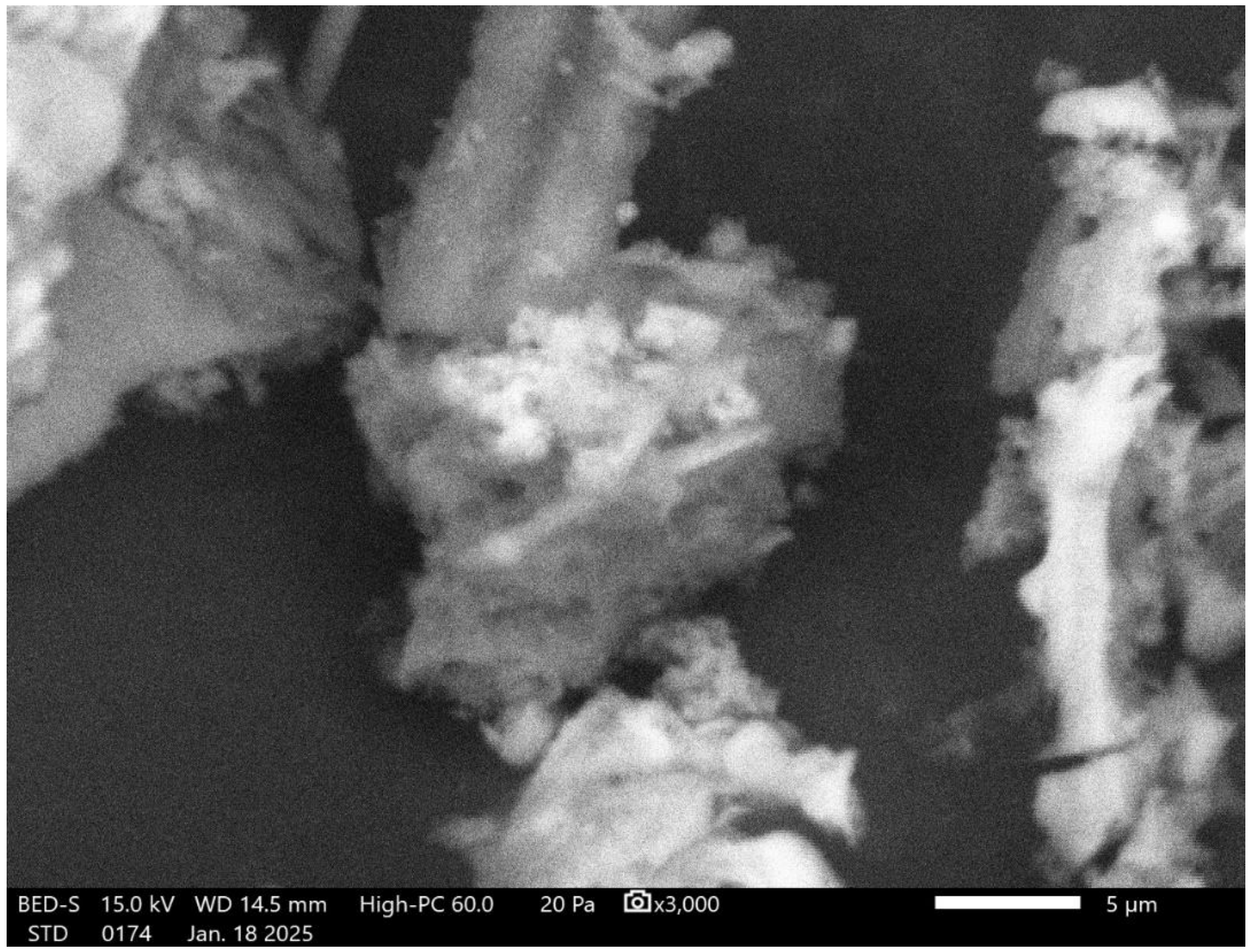
**Corn Cob (CC)**	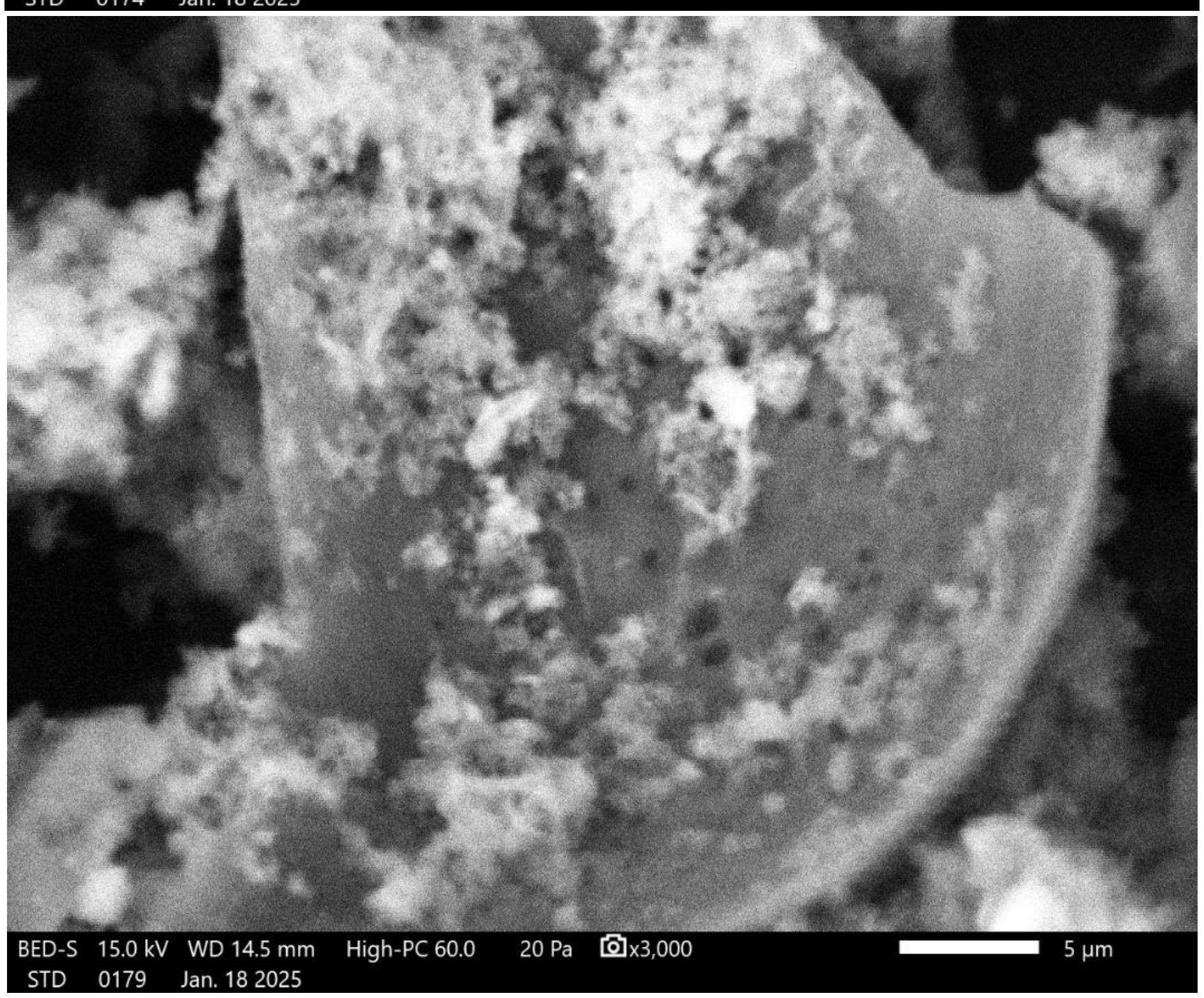

Water Hyacinth (WH)

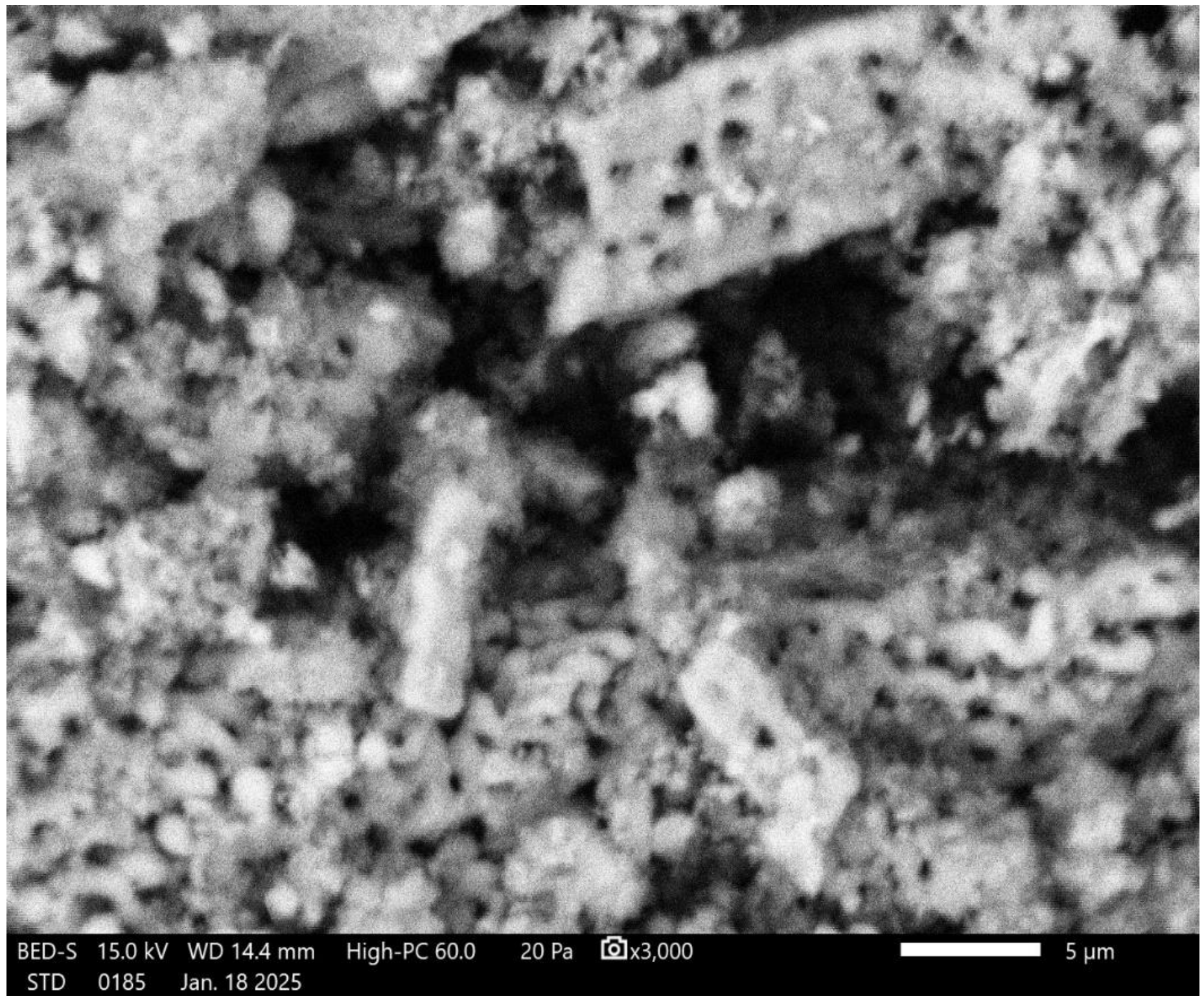

Figure S1. Representative FESEM images of the **(a)** OP, **(b)** BP, **(c)** RS, **(d)** CC, and **(e)** WH activated biomass-derived carbonaceous, respectively.

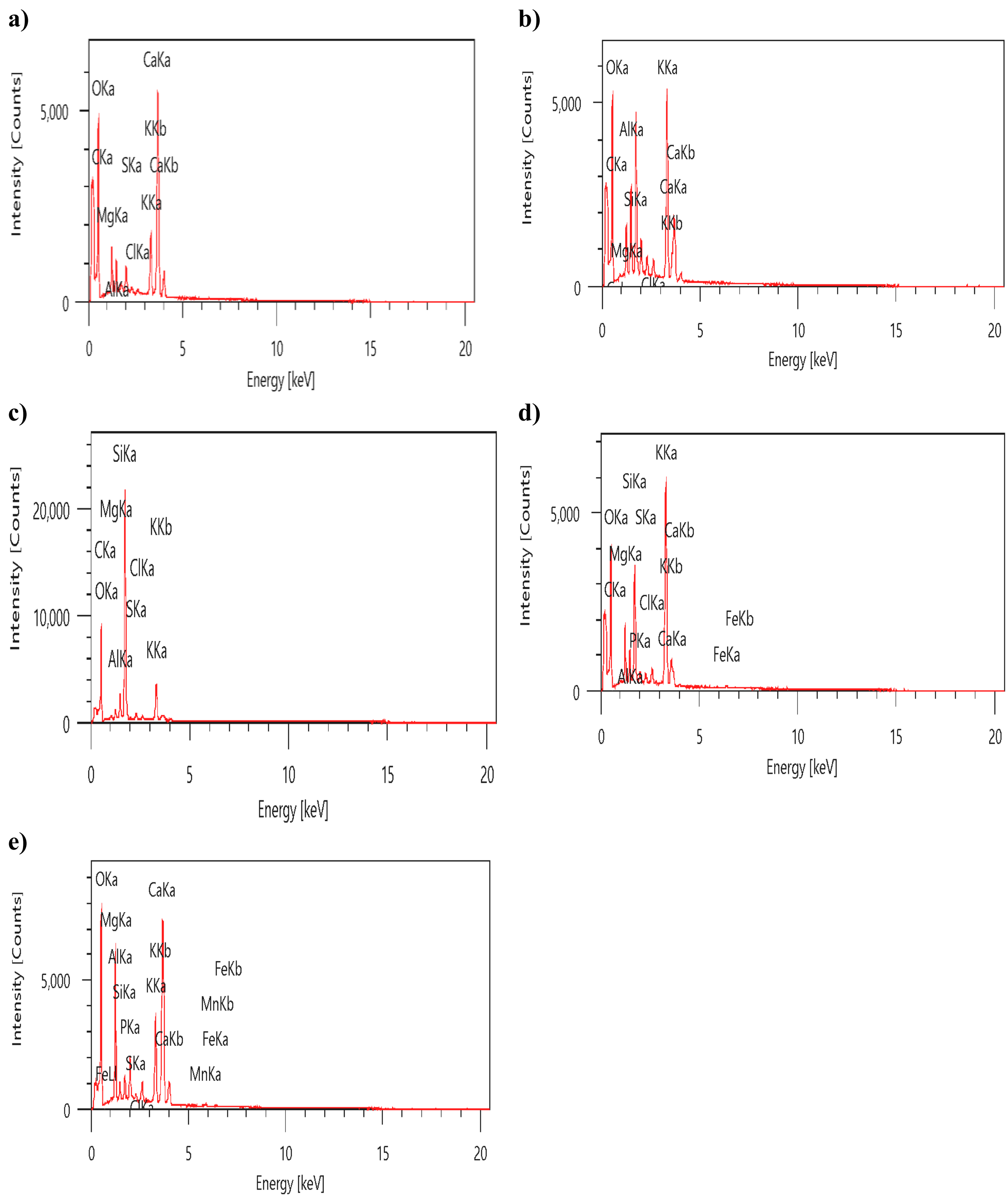

Figure S2. EDX analysis for (a) OP, (b) BP, (c) RS, (d) WH and (e) CC.

<table>
<tr><th>Activated biochar</th><th></th></tr>
<tr><td>Orange Peel (OP)</td><td>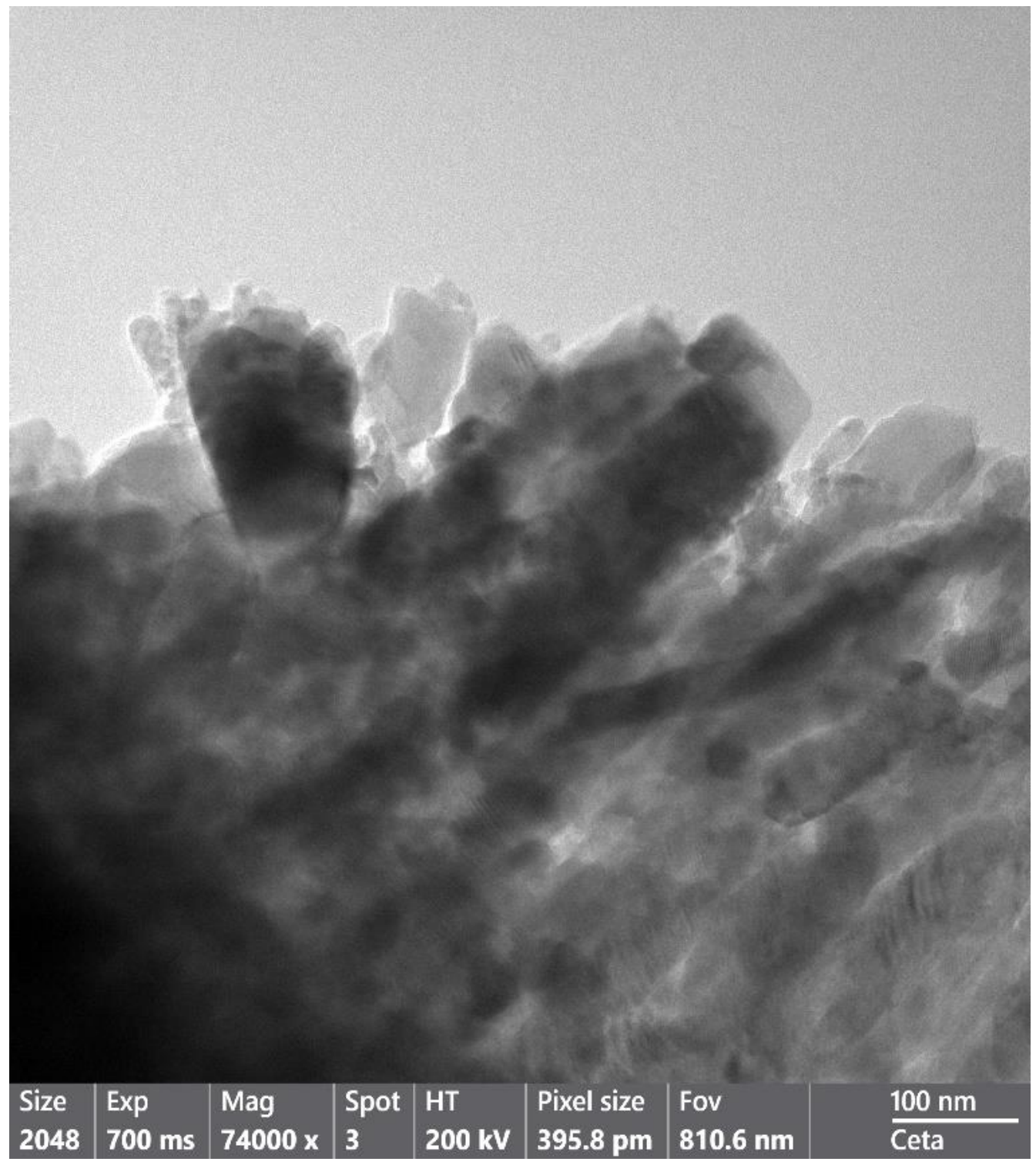
</td></tr>
<tr><td>Banana Peel (BP)</td><td>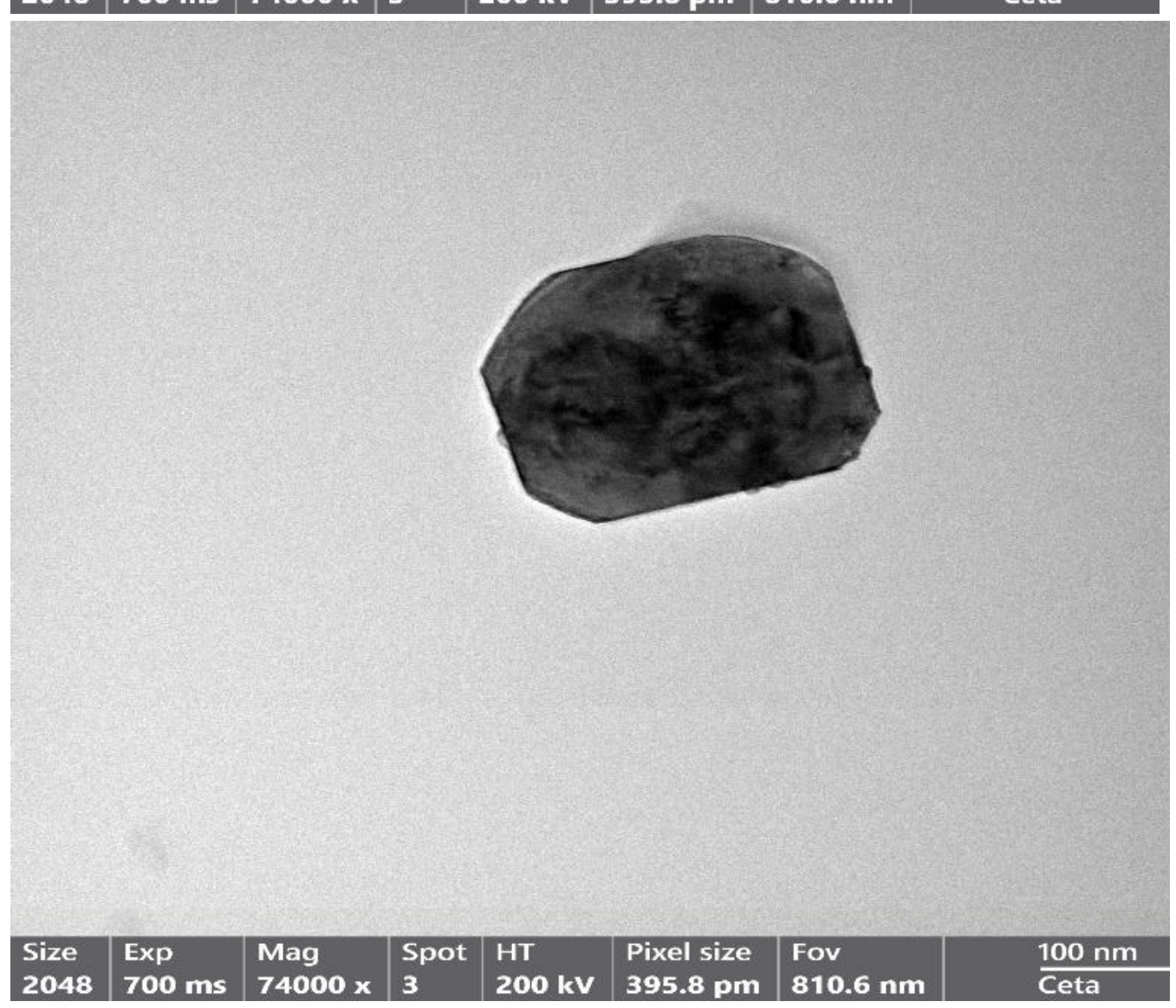
</td></tr>
</table>

Rice Straw (RS)

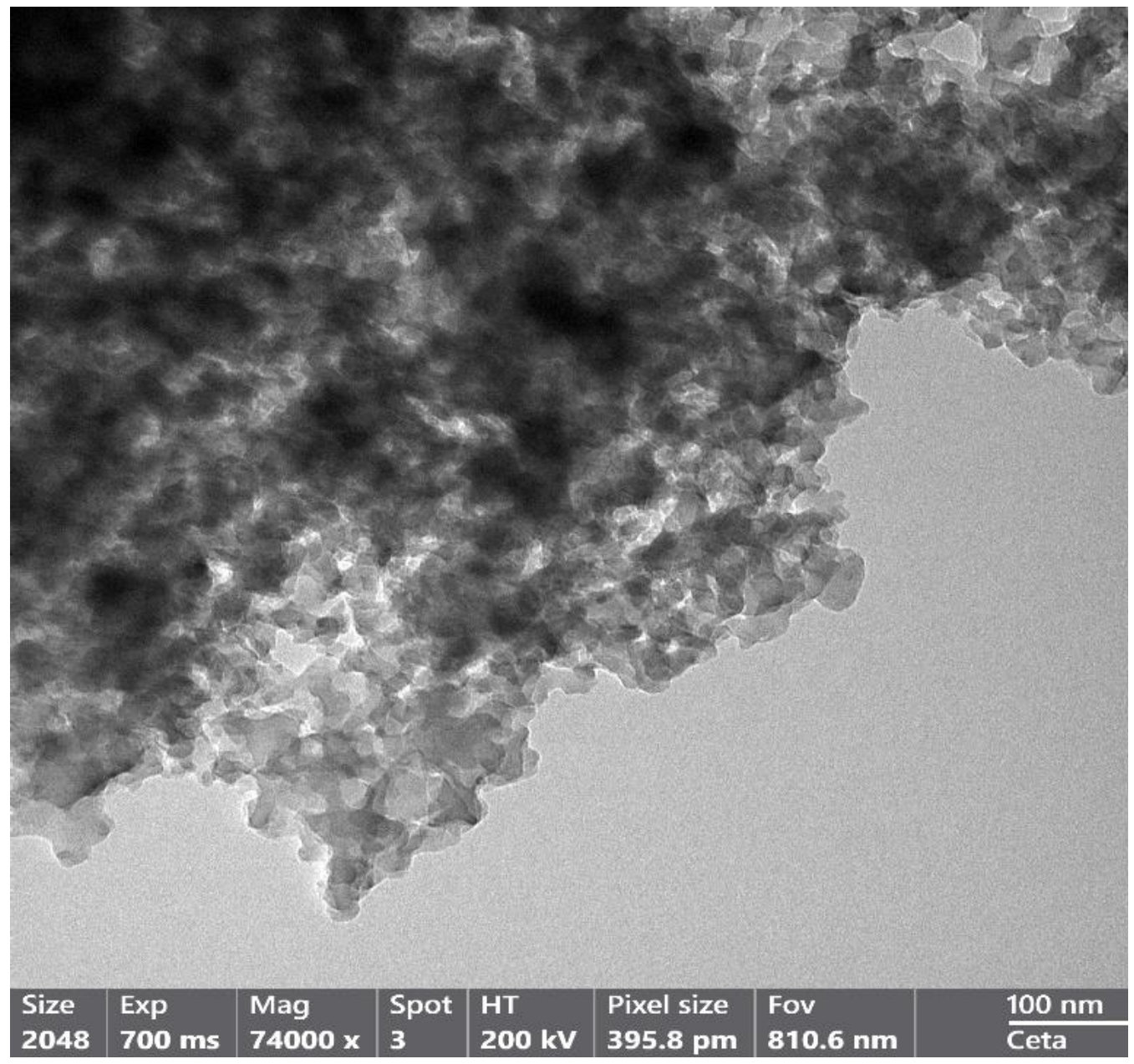

Corn Cob (CC)

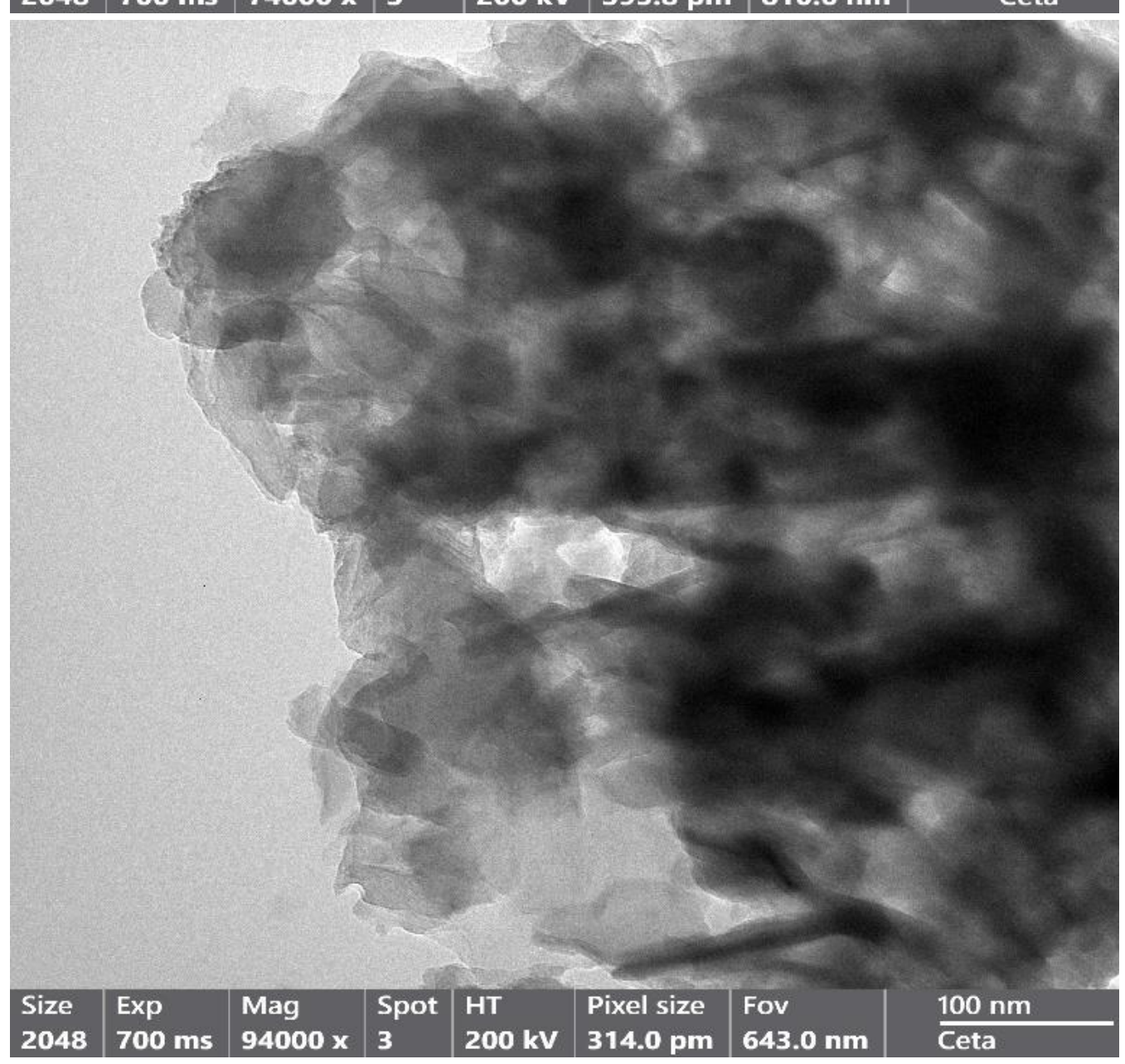

Water Hyacinth (WH)

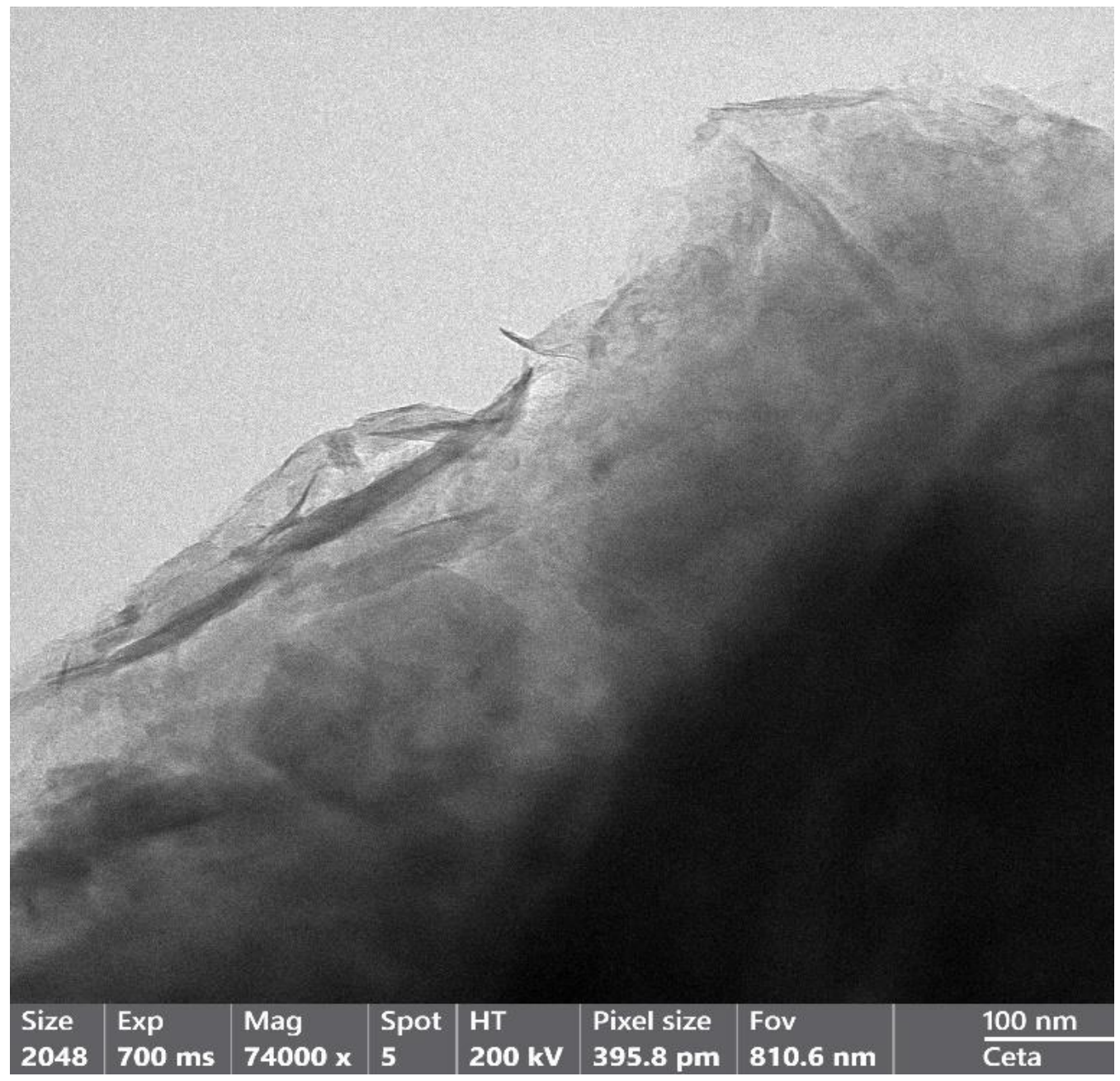

Figure S3. HRTEM images of OP, BP, RS, CC, and WH biochar, respectively.

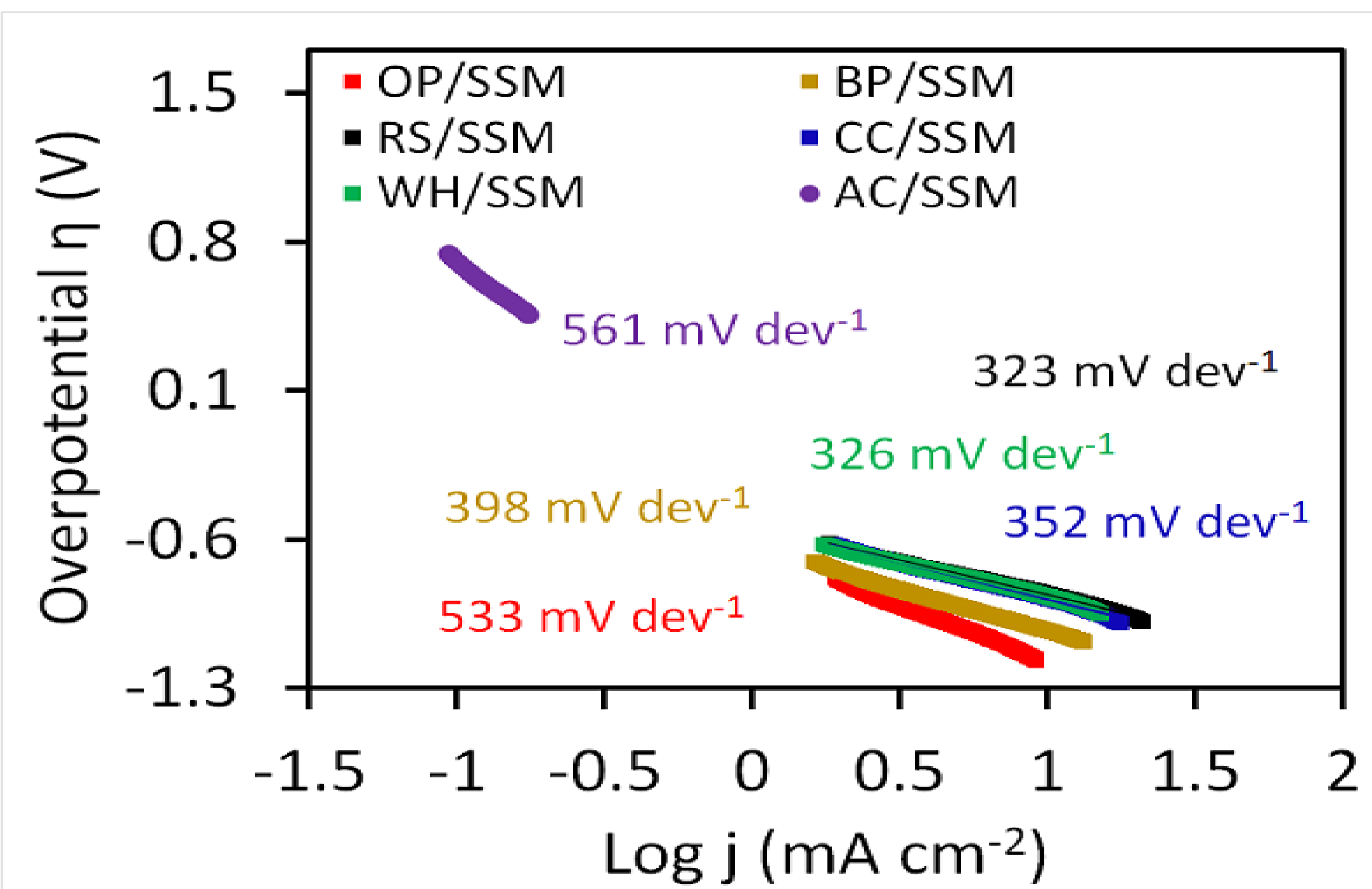

Figure S4. Corresponding Tafel plots with the corresponding Tafel slopes (mV dec^{-1}) for OP/SSM, BP/SSM, RS/SSM, CC/SSM and WH/SSM electrodes in neutral pH.

a) **b)**

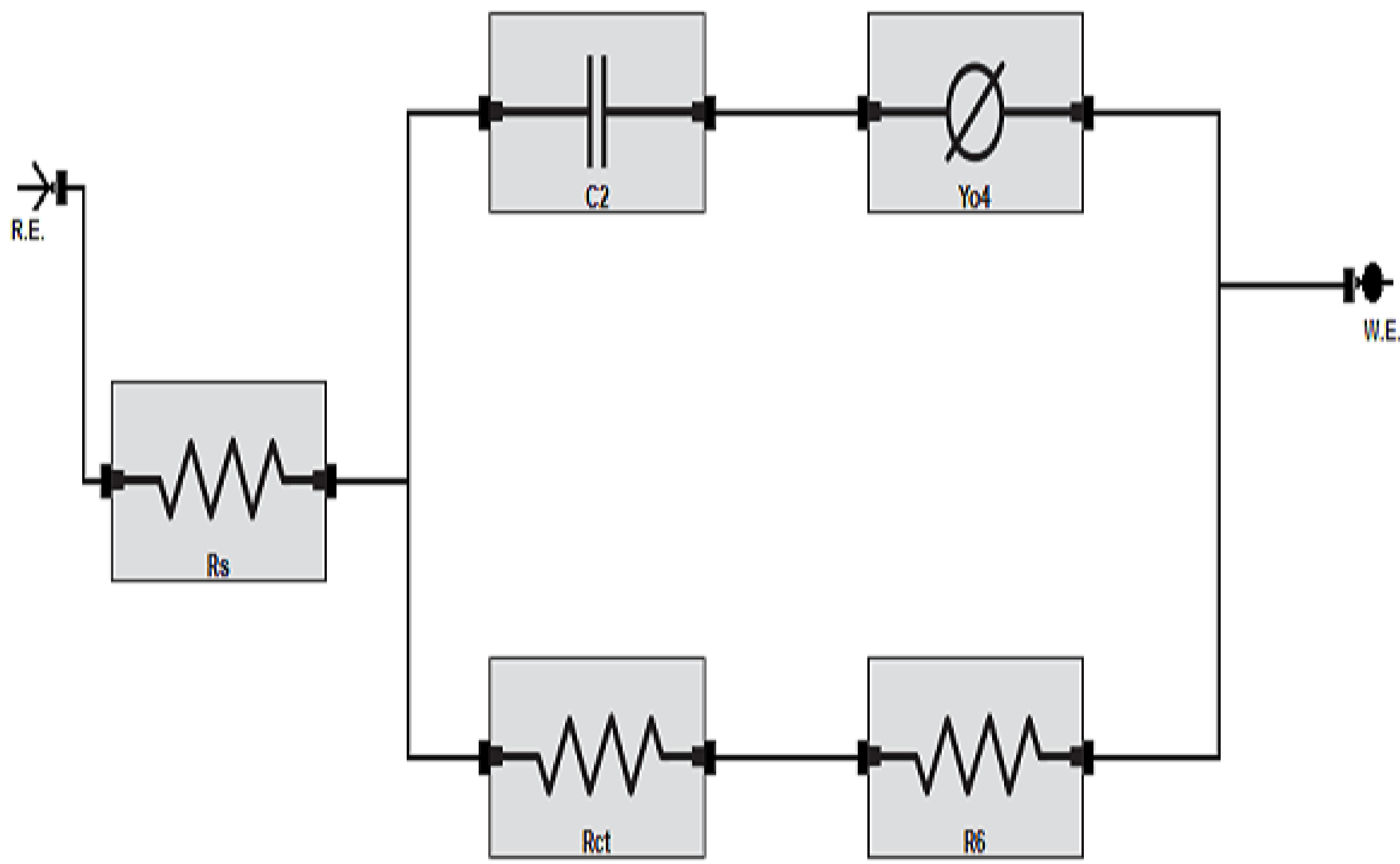

Figure S5. The equivalent fitted circuit.

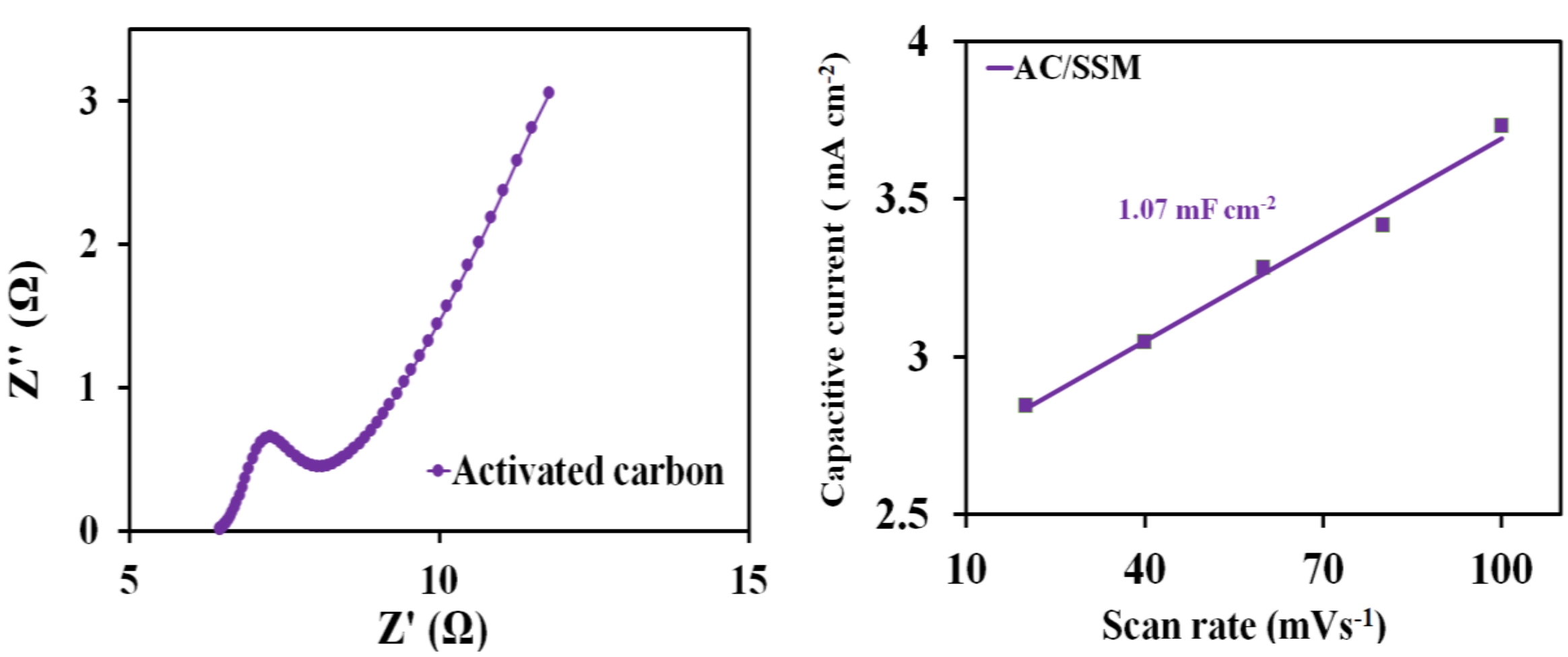

Figure S6. (a) EIS and (b) Cdl plots (linear relationship of charging current density against the applied scan rate ranging from 20-100 mV s^{-1}) for AC/SSM electrodes in neutral electrolyte pH as the control sample.

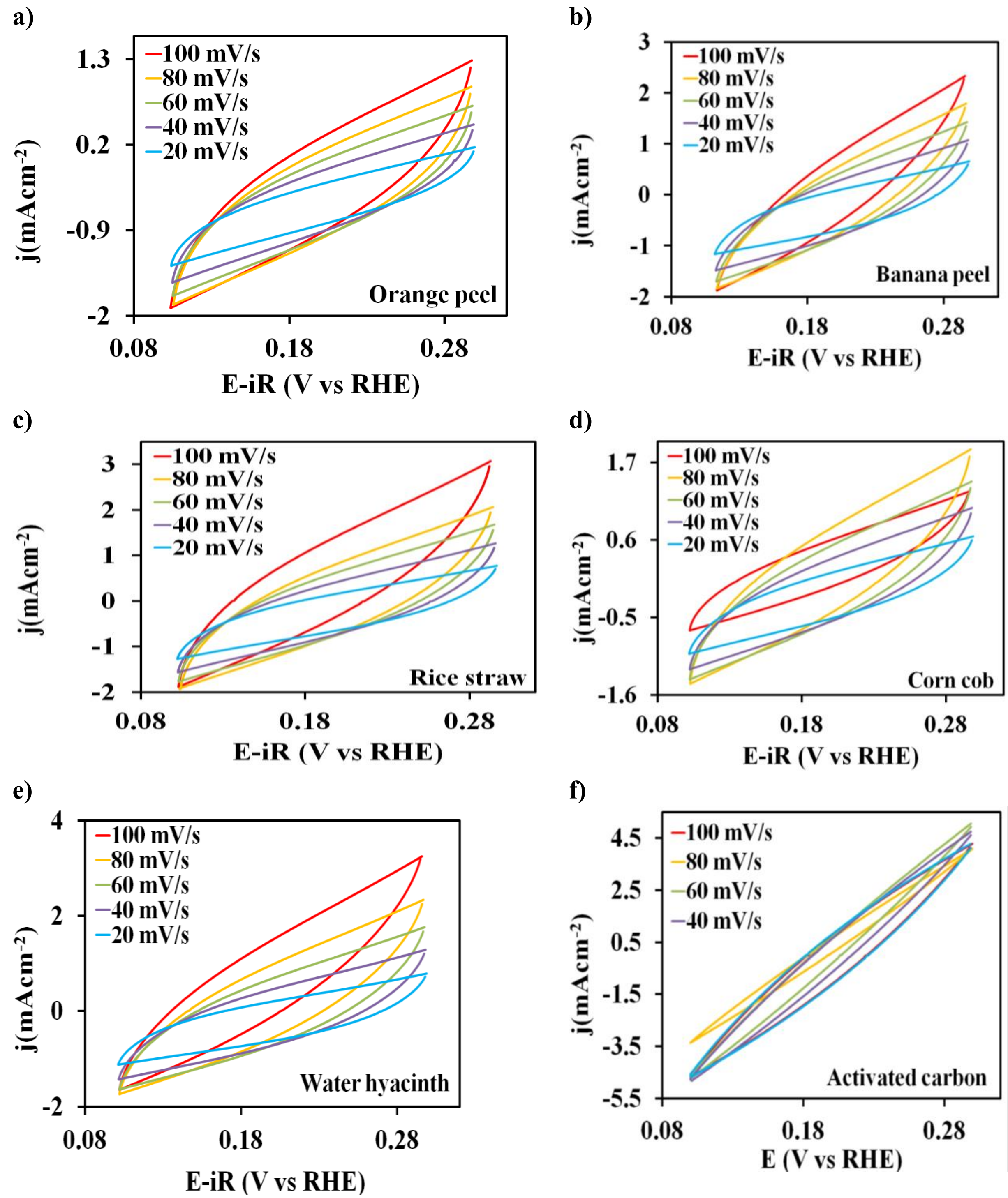

Figure S7. CV curves of the (a) OP/SSM, (b) BP/SSM, (c) RS/SSM, (d) WH/SSM, (e) CC/SSM and (f) AC/SSM at the potential region of -0.31 to -0.51V (versus RHE) and various scan rates (20-100 mV s^{-1}) at the Non-Faradaic region under neutral pH electrolyte (0.1M PBS).

Table S1. Atomic weight percentages of the prepared activated biochar.

Atomic%					
**Element**	**Orange peel**	**Banana Peel**	**Rice straw**	**Corn Cob**	**Water hyacinth**
**C**	14.68±0.27	16.27±0.34	4.13±0.23	15.12±0.35	0.82±0.12
**O**	63.21±1.00	55.04±0.83	58.95±0.68	55.73±0.96	66.85±0.84
**Mg**	2.44±0.09	2.24±0.08	1.01±0.05	3.51±0.11	8.10±0.13
**Al**	1.26±0.06	3.27±0.08	2.67±0.07	1.68±0.07	0.91±0.04
**Si**	0.21±0.02	5.89±0.11	24.78±0.20	5.86±0.12	1.00±0.04
**P**	1.07±0.05	1.62±0.06	0.25±0.03	0.66±0.04	2.10±0.06
**S**	0.22±0.02	0.81±0.04	0.73±0.04	0.49±0.03	0.29±0.02
**Cl**	0.20±0.02	0.79±0.04	0.52±0.03	0.79±0.04	0.29±0.02
**K**	3.23±0.08	10.10±0.14	6.23±0.11	14.61±0.19	0.93±0.04
**Ca**	13.48±0.18	3.97±0.10	-	1.10±0.06	5.12±0.09
**Fe**	-	-	-	0.21±0.04	0.20±0.03

Table S2. Summary of electrochemical results for HER.

AB/SSM	η_{10} (mV)	Tafel (mV dec^{-1})	j_0 (mAcm^{-2})	ECSA (cm^2)	CDL (mFcm^{-2})	Rct (Ω)	Rs (Ω)
OP/SSM	593	496	1.011×10^{-6}	3.2	0.8	2.57	23.76
BP/SSM	514	413	1.010×10^{-6}	3.24	0.81	2.12	14.94
RS/SSM	432	239	1.013×10^{-5}	5.24	1.31	1.13	13.66
WH/SSM	435	285	1.012×10^{-5}	5.0	1.52	2.01	10.14
CC/SSM	436	245	1.133×10^{-5}	3.84	0.960	1.97	12.97
AC/SSM	600	581	1.015×10^{-6}	42.8	1.07	1.09	6.60

Table S3. Fitting quality parameters for the EIS equivalent circuit model.

AB/SSM	Yo4	a5	C2	Rct (Ω)	Rs (Ω)
OP/SSM	1.66×10^{-3}	0.3918	3.04×10^{-3}	2.57	23.76
BP/SSM	1.74×10^{-3}	0.3689	7.52×10^{-3}	2.12	14.94
RS/SSM	1.71×10^{-3}	0.4978	0.4279	1.13	13.66
WH/SSM	1.91×10^{-3}	0.3909	8.73×10^{-3}	2.01	10.14
CC/SSM	1.12×10^{-3}	0.5529	133.8026	1.97	12.97
AC/SSM	1.61×10^{-3}	0.3661	1.93×10^{-4}	1.09	6.60
